# Unexpected Rearrangement of *N*-Allyl-2-phenyl-4,5-Dihydrooxazole-4-Carboxamides to Construct Aza-Quaternary Carbon Centers

**DOI:** 10.3390/molecules24244495

**Published:** 2019-12-08

**Authors:** Goyeong Choi, Seoyoung Jo, Juyeon Mun, Yonguk Jeong, Seok-Ho Kim, Jong-Wha Jung

**Affiliations:** 1 College of Pharmacy, Research Institute of Pharmaceutical Sciences, Kyungpook National University, Daegu 41566, Korea; qlgudtmfkqmf@naver.com (G.C.); tjdud117@naver.com (S.J.); wndus2604@naver.com (J.M.); nemo946@naver.com (Y.J.); 2 Department of Pharmacy, College of Pharmacy and Institute of Pharmaceutical Sciences, CHA University, Gyeonggi-do 11160, Korea; ksh3410@cha.ac.kr

**Keywords:** rearrangement, aza-quaternary carbon center, ring expansion

## Abstract

The unexpected rearrangement of *N*-allyl-2-phenyl-4,5-dihydrooxazole-4-carboxamides in the presence of LiHMDS has been found. The key features are: (1) the net reaction consisted of 1,3-migration of the *N*-allyl group, (2) the rearrangement produced a congested aza-quaternary carbon center, (3) both cyclic and acyclic substrates underwent the unexpected rearrangement to afford products in moderate to high yields, and (4) the reaction seemed to be highly stereoselective. In addition, a plausible mechanism has been discussed.

## 1. Introduction

Rearrangements are highly defined and reliable chemical transformations in terms of efficiency and atom economy. Unlike other chemical reactions, rearrangement involves structural reorganization. This facilitates the construction of congested carbon centers in a highly stereo-controlled fashion. Numerous methods, protocols, and applications have been developed for rearrangement reactions, [3,3]-sigmatropic rearrangement being a textbook example [1,2,3,4]. Among the driving forces that have been discovered or developed to facilitate such rearrangements, the accelerating effects of various substituents continue to attract attention [5,6,7,8].

**Figure 1:**
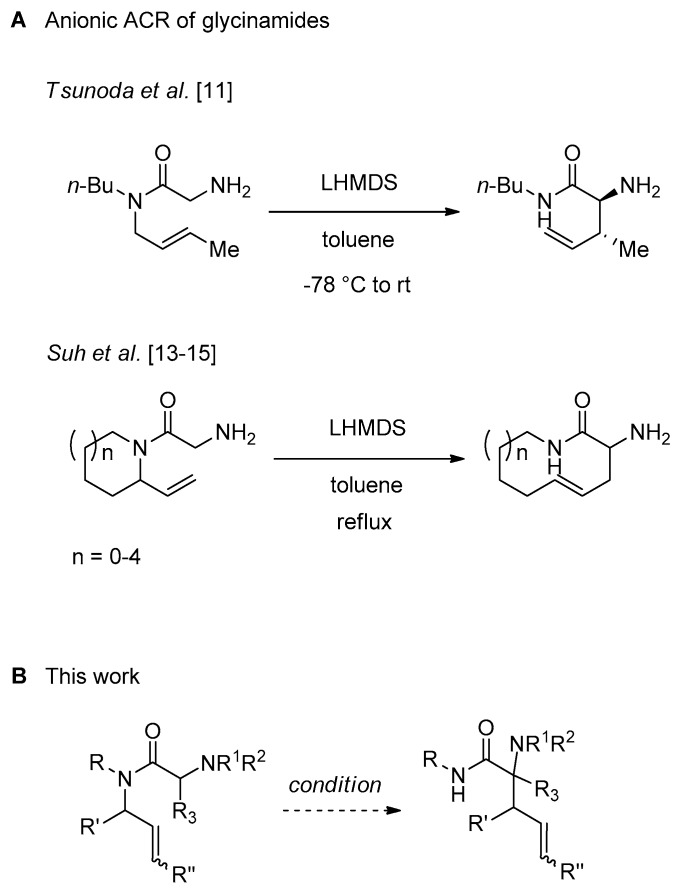
(**A**) Anionic aza-Claisen rearrangement (ACR) of glycinamides; (**B**) this work.

In our research on the synthesis of bioactive alkaloids and small molecules, we became interested in the accelerating effects of substituents in an aza-Claisen rearrangement (ACR) [2]. Much of the effort in the development of ACR has focused on cationic or zwitterionic ACR [9]. Anionic ACR is relatively unexplored, possibly because its activation energy is large [10]. Rate acceleration with the ionic involvement of a neighboring functional group could be utilized to overcome the activation energy barrier of anionic ACR [2]. For instance, Tsunoda et al. [11,12] employed glycinamide for amide-enolate-induced ACR using an acyclic precursor, while Suh et al. [13,14,15] did so with a cyclic precursor (Figure 1A). Rearrangement acceleration in those cases could be attributed to an electronic effect, a chelation effect, or both [11]. Interestingly, those methods have not been expanded for the construction of congested aza-quaternary carbon centers, even though a great deal of effort has been devoted to constructing nitrogen-bearing quaternary centers through molecular rearrangement [16]. We thus became interested in the synthesis of compounds with aza-quaternary carbon centers by accelerating such rearrangements. During our research program, we observed an unexpected rearrangement reaction of *N*-allyl-2-phenyl-4,5-dihydrooxazole-4-carboxamides to construct aza-quaternary carbon centers (Figure 1B). Herein we report the rearrangement reaction, including its scope and limitations.

## 2. Results and Discussion

### 2.1. Substrate Screening and Optimization of Reaction Conditions

We envisioned employing amide-enolate-induced ACR for the construction of congested aza-quaternary carbon centers using glycinamide substrates. We initially evaluated glycinamides as potential substrates for ACR (Table 1, Entries 1–5). Unfortunately, our attempts with glycinamide **1a**, which had a free amine functional group, did not produce the desired ACR product (**2**). Unlike the results reported by Tsunoda et al. and Suh et al. [11,12,13,14,15], our substrate (**1a**) decomposed to unidentified compounds over time (Table 1, Entries 1 and 2). We also tried protecting the glycinamide with various protecting groups (**1b–e**), including Fmoc (Table 1, Entry 3), Boc (Table 1, Entry 4), Cbz (Table 1, Entry 5), and phthalimide (Table 1, Entry 6). However, these attempts failed to provide the desired ACR product (**2**) again. We continued our experiment using a serinamide derivative, 2-phenyl-4,5-dihydrooxazole-4-carboxamide (**1f**, Entries 7–10). To our satisfaction, a product was obtained under the standard amide-enolate-induced ACR conditions [2] in a reasonable yield (Table 1, Entry 7). The reaction was even successful at room temperature (Table 1, Entry 8). We initially thought we obtained the desired ACR product (**2**). To our surprise, ^1^H NMR spectroscopy revealed terminal vinyl protons instead of the expected internal olefin protons. After carefully analyzing the spectral data, the compound was found to be an unexpected rearrangement product (**3**) rather than the ACR product (**2**). After careful analysis of ^1^H NMR spectrum, the rearrangement product (**3**) appeared to be a single diastereomer in a view of relative stereochemistry, even though it could not be determined. To the best of our knowledge, this type of rearrangement has never been reported. After evaluating a different solvent (Table 1, Entries 8 and 9) and base (Table 1, Entries 9 and 10), we had our optimized condition (Table 1, Entry 9).

### 2.2. Preparation of Substrates

**Scheme 1:**
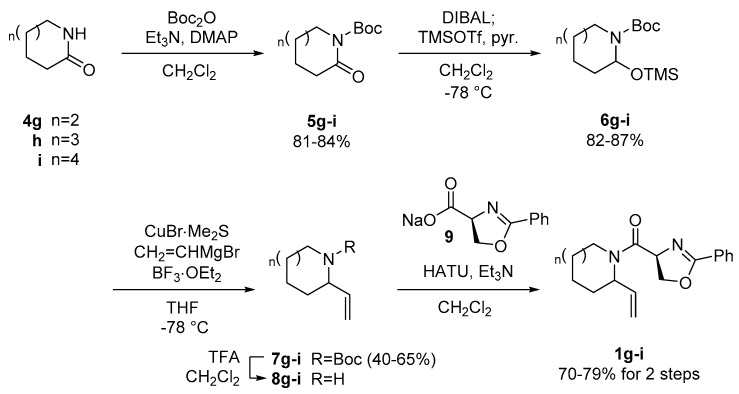
Preparation of serinamides **1g–i**.

Next, we explored the scope of the reaction using various substrates to determine whether the unexpected rearrangement could be generalized. The requisite serinamide derivatives were prepared as shown in Scheme 1. Seven- to nine-membered vinyl azacycles (**1g–i**) were synthesized from the corresponding lactams (**4g–i**) as previously reported [17]. Briefly, partial reduction of the Boc-protected lactams (**5g–i**) and consecutive trimethylsilyl (TMS) trapping yielded *N,O*-acetal TMS ethers (**6g–i**). The *N*-acyliminium ion precursors (**6g–i**) were treated with vinyl Grignard reagent and copper salt in the presence of BF_3_ as a Lewis acid to give vinyl azacycles (**7g-i**), which were then treated with trifluoroacetic acid (TFA) for deprotection. Finally, the resulting TFA salts (**8g–i**) were cross-coupled with sodium carboxylate (**9**) to afford the desired serinamides (**1g–i**) as diastereomeric mixtures. It was worth noting that after screening a series of coupling agents, 1-[bis(dimethylamino)methylene]-1H-1,2,3-triazolo[4,5-b]pyridinium 3-oxide hexafluorophosphate (HATU) was the most efficient reagent in terms of chemical yield. The required sodium carboxylate (**9**) was prepared from *L*-serine methyl ester according to a reported procedure [18].

**Scheme 2:**
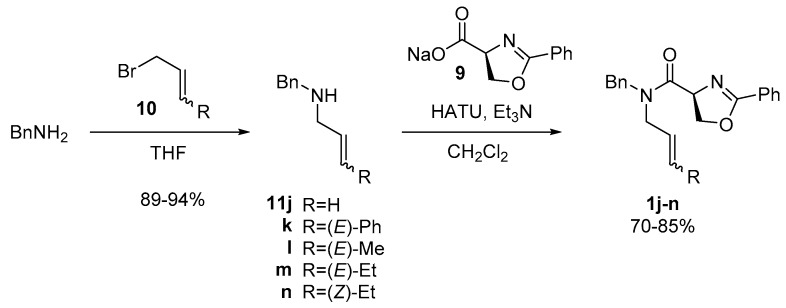
Preparation of serinamides **1j–n**.

To synthesize the acyclic serinamide derivatives (**1j–n**), secondary amines with allyl or substituted allyl groups were first prepared from commercially available benzyl amine and the corresponding alkyl bromides (**10**, Scheme 2). The resulting secondary amines (**11j–n**) were cross-coupled with sodium carboxylate (**9**) to afford the desired serinamide derivatives (**1j–n**) in moderate to high yields.

### 2.3. Reaction Scope

Reacting the seven- to nine-membered cyclic substrates (**1g–i**) in the presence of LiHMDS afforded the expected rearrangement products (**3g–i**) in moderate to high yields at room temperature (Table 2, Entries 2–4). All of the products (**3g–i**) appeared to be single diastereomers in the ^1^H NMR spectra. An acyclic substrate with a terminal olefin (**1j**) also provided the expected product (**3j**) within a very short period of time (Table 2, Entry 5). Unlike the reactions that generated the cyclic substrates (**3f–i**), ACR could not be ruled out as a plausible mechanism in this case. We next performed the reactions with acyclic substrates using substituted olefins. However, the reaction between the acyclic substrate and the phenyl-substituted olefin **1k** was slower. Only trace amounts of unidentified products were obtained after many hours at room temperature. We thus tried reacting **1k–n** by refluxing them in the solvent, which effectively generated the expected rearrangement products (**3k–n**) after 12 h (Table 2, Entries 7–10). In this case, the ^1^H NMR spectrum of each product (**3k–n**) clearly showed a pair of internal olefin protons. It was noted that the internal olefin geometry was conserved after the reactions (Table 2, Entries 8 and 9). A larger substituent reduced the chemical yield, possibly through steric interactions. Based on the results of reacting acyclic substrates with substituted olefins, we concluded that the reaction between acyclic substrates and terminal olefins might proceed by the same mechanism as the others rather than ACR.

### 2.4. Proposed Mechanism

**Figure 2:**
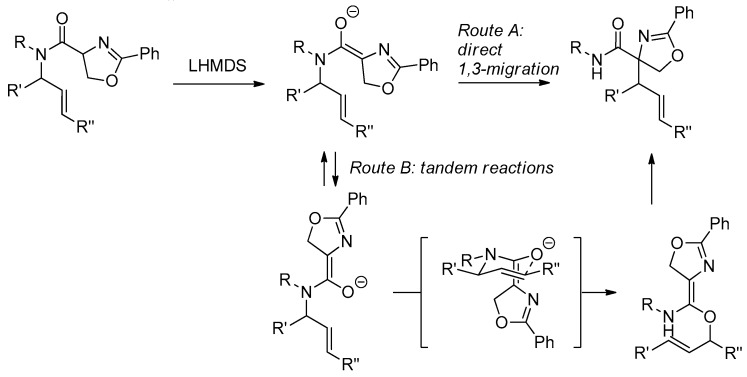
Plausible mechanism for the unexpected rearrangement.

Plausible mechanism for the unexpected rearrangement is shown in Figure 2. One mechanism (Route A) is 1,3-migration of the *N*-allyl group (Route A), while the other involves tandem reactions (Route B). To investigate the direct 1,3-migration mechanism which is similar to 1,2-migration of Stevens rearrangement [19,20,21,22] we performed the reaction with substrates bearing *N*-propyl (saturated) or *N*-benzyl groups. This failed to provide the expected products, so rearrangement more likely occurred through tandem reactions even though we could not isolate any of the possible intermediates of tandem reactions. Since the geometry of the internal olefins was conserved (Table 2, Entries 8 and 9), the tandem reactions appeared to be highly stereo-controlled. Further studies are needed to confirm the unexpected reaction mechanism.

## 3. Materials and Methods

### 3.1. General Information

Unless noted otherwise, all starting materials and solvents were used as obtained from commercial suppliers (Aldrich, Yongin, Korea) without further purification. Organic solvents used in this study were dried over appropriate drying agents and distilled prior to use. Thin layer chromatography was carried out using Merck silica gel 60 F_254_ plates, and flash chromatography was performed automatically with Biotage Isolera or manually using Merck silica gel 60 (0.040–0.063 mm, 230–400 mesh, Seoul, Korea). ^1^H and ^13^C NMR spectra were recorded using JEOL-500 and BRUKER (Seongnam, Korea) AVANCE-500. ^1^H and ^13^C NMR chemical shifts are reported in parts per million (ppm) relative to TMS, with the residual solvent peak used as an internal reference. Low and high resolution mass spectra were obtained with JEOL JMS-700 instrument (Seoul, Korea) and Agilent Q TOF 6530 (Seoul, Korea). ^1^H NMR data were reported in the order of chemical shift, multiplicity (br, broad signal; s, singlet; d, doublet; t, triplet; q, quartet; quint, quintet; m, multiplet and/or multiple resonances), number of protons, and coupling constant in hertz (Hz). ^1^H and ^13^C NMR spectra of compounds **1f-n** and **3f-n** are available in Supplementary Materials.

### 3.2. Experimental

#### 3.2.1. Representative Procedure: Synthesis of Compound **1g**

TFA (0.383 mL, 5.00 mmol) was added to a solution of compound **7g** (113 mg, 0.502 mmol) [17] in CH_2_Cl_2_ (2.5 mL). The reaction mixture was stirred till completion. Then, volatiles were removed in vacuo to afford crude compound **8g**. Compound **8g** was used for next reaction without further purification. To a stirred solution of the above compound **8g** in CH_2_Cl_2_ (2.5 mL), compound **9** (148 mg, 0.751 mmol) [18], HATU (285 mg, 0.751 mmol) and Et_3_N (0.139 mL, 1.00 mmol) were added. The reaction mixture was stirred overnight. The resulting mixture was quenched with water, and extracted with CH_2_Cl_2_. The organic extracts were dried over anhydrous MgSO_4_. After filtration, the filtrate was concentrated under reduced pressure. The residue was purified by SiO_2_ chromatography to afford the desired product **1g** (118 mg, 0.396 mmol).

#### 3.2.2. (2-Phenyl-4,5-dihydrooxazol-4-yl)(2-vinylpiperidin-1-yl)methanone (**1f**)

Yield 95% for 2 steps, a colorless oil; ^1^H NMR (300 MHz, CDCl_3_) δ 7.96–7.89 (m, 2H), 7.49-7.30 (m, 3H), 5.96–5.76 (m, 1H), 5.40–4.88 (m, 5H), 4.49–4.40 (m, 2H), 3.25 and 2.75 (m, 1H), 2.04–1.43 (m, 6H); ^13^C NMR (500 MHz, CDCl_3_) δ 167.8, 164.5, 137.05, 136.4, 131.4, 128.4, 128.2, 127.5, 117.2, 116.2, 69.3, 69.2, 69.1, 67.6, 67.2, 54.4, 50.8, 42.0, 38.2, 29.8, 29.3, 28.4, 26.3, 25.8, 25.2, 19.7, 19.6; HRMS (EI+) calcd for C_17_H_20_N_2_O_2_ [M]^+^: 284.1525, found: 284.1526.

#### 3.2.3. (2-Phenyl-4,5-dihydrooxazol-4-yl)(2-vinylazepan-1-yl)methanone (**1g**)

Yield 79% for 2 steps, a colorless oil; ^1^H NMR (500 MHz, CDCl_3_) δ 7.93–7.92 (m, 2H), 7.48–7.44 (m, 1H), 7.40–7.37 (m, 2H), 5.75–5.69 (m, 1H), 5.31–4.90 (m, 5H), 4.59–4.14 (m, 2H), 3.14 and 2.75 (m, 1H), 2.42–1.22 (m, 8H); ^13^C NMR (500 MHz, CDCl_3_) δ 169.0, 168.6, 138.9, 133.1, 131.3, 128.5, 128.5, 128.4, 128.4, 114.1, 114.0, 59.2, 42.6, 34.3, 33.7, 29.7, 27.1, 26.6, 25.6, 24.6; HRMS (EI+) calcd for C_18_H_22_N_2_O_2_ [M]^+^: 298.1681, found: 298.1684.

#### 3.2.4. (2-Phenyl-4,5-dihydrooxazol-4-yl)(2-vinylazocan-1-yl)methanone (**1h**)

Yield 70% for 2 steps, a colorless oil; ^1^H NMR (500 MHz, CDCl_3_) δ 7.92–7.90 (m, 2H), 7.47 (t, *J* = 3.0 Hz, 1H), 7.41–7.38 (m, 2H), 5.83–5.77 (m, 1H), 5.32–4.92 (m, 6H), 4.48–4.38 (m, 1H), 3.94–3.79 (m, 2H), 2.93–2.87 (m, 1H), 2.05–1.25 (m, 10H); ^13^C-NMR(500 MHz, CDCl_3_) δ 168.9, 138.9, 137.9, 131.5, 128.5, 128.4, 128.4, 128.3, 128.3, 128.2, 114.0, 68.9, 67.4, 58.7, 42.5, 29.7, 27.0, 26.6, 26.1, 26.0, 25.6, 24.5; HRMS (EI+) calcd for C_19_H_24_N_2_O_2_ [M]^+^: 312.1838, found: 312.1841.

#### 3.2.5. (2-Phenyl-4,5-dihydrooxazol-4-yl)(2-vinylazonan-1-yl)methanone (**1i**)

Yield 72% for 2 steps, a colorless oil; ^1^H NMR (500 MHz, CDCl_3_) δ 7.92–7.90 (m, 2H), 7.46–7.43 (m, 1H), 7.39–7.36 (m, 2H), 5.78–5.71(m, 1H), 5.37–5.24 (m, 3H), 4.98–4.93 (m, 1H), 4.47–4.39 (m, 1H), 3.87–3.83 (m, 1H), 2.80–2.75 (m, 2H), 2.15–1.20 (m, 14H); ^13^C NMR (500 MHz, CDCl_3_) δ 169.7, 164.4, 139.0, 131.5, 129.8, 128.5, 128.4, 128.3, 128.3, 127.6, 114.7, 114.4, 77.6, 69.1, 67.8, 59.4, 44.7, 32.0, 30.2, 29.7, 29.7, 29.4, 27.6, 26.9, 26.0, 25.7, 23.5, 22.7, 14.1; HRMS (EI+) calcd for C_20_H_26_N_2_O_2_ [M]^+^: 326.1994, found: 326.1998.

#### 3.2.6. *N*-allyl-*N*-benzyl-2-phenyl-4,5-dihydrooxazole-4-carboxamide (**1j**)

Yield 85%, a colorless sticky oil; ^1^H NMR 300 MHz, CDCl_3_) δ 7.90–7.81 (m, 2H), 7.42–7.17 (m, 8H), 5.87 (m, 1H), 5.20–4.93 (m, 4H), 4.79 (d, *J* = 15.8 Hz, 1H), 4.55 (m, 1H), 4.40 (d, *J* = 15.2 Hz, 2H), 4.10 (m, 1H); ^13^C NMR (500 MHz, CDCl_3_) δ 169.4, 169.2, 164.6, 164.5, 137.0, 133.5, 132.3, 131.3, 128.5, 128.3, 128.2, 128.0, 127.8, 127.2, 127.0, 126.8, 117.2, 116.8, 69.0, 67.4, 67.1, 49.8, 49.0, 48.3, 48.0; HRMS (EI+) calcd for C_20_H_20_N_2_O_2_ [M]^+^: 320.1525, found: 320.1528.

#### 3.2.7. (*E*)-*N*-benzyl-*N*-cinnamyl-2-phenyl-4,5-dihydrooxazole-4-carboxamide (**1k**)

Yield 75%, a colorless sticky oil; ^1^H NMR (500 MHz, CDCl_3_) δ 8.01–7.99 (m, 1H), 7.96–7.94 (m, 1H), 7.54–7.24 (m, 13H), 6.56–6.46 (m, 1H), 6.35–6.17 (m, 1H), 5.29–4.03 (m, 5H); ^13^C NMR (500 MHz, CDCl_3_) δ 169.7, 169.6,165.0,164.9, 137.2,136.6, 136.4, 133.2, 132.2, 131.6, 131.6, 128.8, 128.7, 128.6, 128.5, 128.3, 128.3, 128.2, 127.9, 127.7, 127.5, 127.5, 127.4, 127.4, 127.0, 126.5, 126.4, 125.1, 124.1, 69.3, 69.3, 67.7, 67.6, 50.1, 48.9, 48.7, 47.9; HRMS (EI+) calcd for C_26_H_24_N_2_O_2_ [M]^+^: 396.1838, found: 396.1838.

#### 3.2.8. (*E*)-*N*-benzyl-*N*-(but-2-en-1-yl)-2-phenyl-4,5-dihydrooxazole-4-carboxamide (**1l**)

Yield 70%, a colorless sticky oil; ^1^H NMR (500 MHz, CDCl_3_) δ 7.99 (d, *J.* = 11.5 Hz 1H), 7.91 (d, *J* = 7.8 Hz 1H), 7.50–7.20 (m, 8H), 5.66–5.20 (m, 2H), 5.18–4.95 (m, 2H), 4.51–4.41 (m, 2H), 4.11–4.01 (m, 1H), 1.68–1.54 (m, 3H); ^13^C NMR (125 MHz, CDCl_3_) δ 169.5, 169.4, 164.9, 137.4, 137.3, 131.5, 129.3, 129.0, 128.8, 128.7, 128.6, 128.5, 128.4, 128.3, 128.2, 128.1, 128.0, 127.6, 127.5, 127.3, 127.1, 127.0, 126.4, 126.3, 125.4, 125.2, 69.4, 69.3, 67.7, 67.5, 67.4, 50.2, 49.8, 48.7, 48.3, 47.7, 43.8, 42.3, 30.9, 29.7, 17.8, 17.7, 13.1, 12.9; HRMS (EI+) calcd for C_20_H_18_N_2_O_2_ [M]^+^: 334.1681, found: 334.1677.

#### 3.2.9. (*E*)-*N*-benzyl-*N*-(pent-2-en-1-yl)-2-phenyl-4,5-dihydrooxazole-4-carboxamide (**1m**)

Yield 72%, a colorless oil; ^1^H NMR (500 MHz, CDCl_3_) δ 7.97 (d, *J* = 7.2 Hz, 1H), 7.91 (d, *J* = 7.2 Hz, 1H), 7.49–7.44 (m, 1H), 7.41–7.35(m, 3H), 7.30–77.22 (m, 4H), 5.68–5.39 (m, 2H), 5.19–5.01 (m, 2H), 4.86–4.73 (m, 1H), 4.51–4.41 (m, 2H), 4.12–3.83 (m, 1H), 2.11–2.00 (m, 2H), 1.02–0.94 (m, 3H); ^13^C NMR (125 MHz, CDCl_3_) δ 206.8, 169.6, 164.9, 137.4, 136.4, 135.7, 131.5, 128.7, 128.5, 128.3, 128.1, 127.6, 127.5, 127.4, 127.2, 127.0, 124.0, 124.1, 123.1, 69.3, 67.7, 67.4, 49.8, 48.7, 48.4, 47.7, 30.9, 25.3, 13.4; HRMS (EI+) calcd for C_22_H_24_N_2_O_2_ [M]^+^: 348.1838, found: 348.1835.

#### 3.2.10. (*Z*)-*N*-benzyl-*N*-(pent-2-en-1-yl)-2-phenyl-4,5-dihydrooxazole-4-carboxamide (**1n**)

Yield 72%, a colorless sticky oil; ^1^H NMR (500 MHz, CDCl_3_) δ 7.98–7.97 (m, 1H), 7.92–7.90 (m, 1H), 7.50–7.26 (m, 8H), 5.22–4.41 (m, 7H), 4.13–3.99 (m, 1H), 2.10–1.94 (m, 2H), 1.00–0.91 (m, 3H); ^13^C NMR (125 MHz, CDCl_3_) δ 169.5, 169.4, 164.9, 164.8, 137.3,137.3,135.8, 135.6, 131.5,131.5, 128.7, 128.6, 128.5, 128.3, 128.3, 128.1, 127.6, 127.5, 127.3, 127.0, 124.7, 123.6, 67.3, 67.7, 67.5, 50.1, 48.6, 44.0, 42.5, 20.8, 20.6, 14.1; HRMS (EI+) calcd for C_22_H_24_N_2_O_2_ [M]^+^: 348.1838, found: 348.1835.

#### 3.2.11. Representative Procedure: Synthesis of Compound **3f**

To stirred solution of **1f** (292 mg, 1.02 mmol) in benzene (5 mL) was added LHMDS (3.06 mL, 1M in hexane) and stirred for 1 h. The reaction mixture was quenched with aq. NH_4_Cl solution, and extracted with EtOAc. The organic extracts were dried over anhydrous MgSO_4_. After filtration, the filtrate was concentrated under reduced pressure. The residue was purified by SiO_2_ chromatography to afford the desired product **3f** (280 mg, 0.98 mmol). 

#### 3.2.12. 2-Phenyl-12-vinyl-3-oxa-1,7-diazaspiro[4.7]dodec-1-en-6-one (**3f**)

Yield 96%, a colorless oil; ^1^H NMR (300 MHz, CDCl_3_) δ 8.19 (s, 1H), 7.84–7.78 (m, 2H), 7.45–7.33 (m, 3H), 6.15 (s, 1H), 5.90–5.79 (m, 1H), 5.34–5.10 (m, 4H), 4.27 (d, *J* = 14.7 Hz, 1H), 3.03 (t, *J* = 12.3 Hz, 1H), 1.80-1.49 (m, 6H); ^13^C NMR (125 MHz, CDCl_3_) δ 167.4, 165.8, 136.2, 135.5, 133.7, 131.7, 128.4, 127.2,116.8, 103.6, 29.1, 25.6, 19.8; HRMS (EI+) calcd for C_17_H_20_N_2_O_2_ [M]^+^: 284.1525, found: 284.1528.

#### 3.2.13. 2-Phenyl-13-vinyl-3-oxa-1,7-diazaspiro[4.8]tridec-1-en-6-one (**3g**)

Yield 71%, a colorless oil; ^1^H NMR (500 MHz, CDCl_3_) δ 8.06 (s, 1H), 7.82–7.80 (m, 2H), 7.54–7.51 (m, 1H), 7.46–7.44 (m, 2H) 6.16 (s, 1H), 5.86–5.79 (m, 1H), 5.25 (s, 1H), 5.18 (d, *J* = 10.5 Hz, 1H), 5.10 (d, *J* = 17.0 Hz, 1H), 4.75 (s, 1H), 4.13–4.10 (m, 1H), 2.82 (d, *J* =12.5, 1H) 2.20–2.16 (m, 1H), 1.84–1.73 (m, 3H) 1.55–1.24 (m, 5H); ^13^C NMR (125 MHz, CDCl_3_) δ 166.0, 138.4, 134.3, 132.0, 128.7, 127.1, 114.3, 103.9, 60.4, 42.9, 34.9, 30.0, 29.7, 29.4, 26.9, 24.3, 22.7; HRMS (EI+) calcd for C_18_H_22_N_2_O_2_ [M]^+^: 298.1681, found: 298.1679.

#### 3.2.14. 2-Phenyl-14-vinyl-3-oxa-1,7-diazaspiro[4.9]tetradec-1-en-6-one (**3h**)

Yield 76%, a colorless oil; ^1^H NMR (500 MHz, CDCl_3_) δ 7.92 (s, 1H), 7.78 (d, *J* = 7.5 Hz, 2H), 7.52–7.49 (m, 1H), 7.44 (t, *J* = 7.5 Hz, 2H), 6.29 (s, 1H), 5.75–5.63 (m, 1H), 5.13–4.87 (m, 5H), 3.96–3.90 (m, 1H), 2.94–2.88 (m, 1H), 2.15–1.45 (m, 9H), 1.25–1.22 (m, 1H); ^13^C NMR (125 MHz, CDCl_3_) δ 171.3, 167.9, 166.2, 166.1, 138.7, 137.2, 135.0, 132.0, 128.8, 128.8, 127.0, 127.0, 115.3, 115.2, 104.0, 103.3, 61.2, 60.4, 58.4, 42.8, 40.7, 34.3, 30.3, 30.0, 29.2, 26.6, 26.3, 26.1, 26.0, 25.8, 24.2, 24.1, 21.1, 14.2; HRMS (EI+) calcd for C_19_H_24_N_2_O_2_ [M]^+^: 312.1838, found: 312.1842.

#### 3.2.15. 2-Phenyl-15-vinyl-3-oxa-1,7-diazaspiro[4.10]pentadec-1-en-6-one (**3i**)

Yield 77%, a colorless oil; ^1^H NMR (500 MHz, CDCl_3_) δ 7.92 (s, 1H), 7.79 (t, *J* = 7.5 Hz, 2H), 7.55–7.53 (m, 1H), 7.47–7.44 (m, 2H), 6.32 (s, 1H), 5.78–5.70 (m, 2H), 5.18–5.14 (m 2H), 5.06–4.93 (m, 3H), 3.93–3.85 (m, 2H), 2.90–2.80 (m, 1H) 2.09–1.88 (m, 3H), 1.77–1.70 (m, 4H), 1.47–1.42 (m, 3H), 1.27–1.25 (m, 1H); ^13^C NMR (125 MHz, CDCl_3_) δ 168.6, 166.1, 138.7, 134.4, 132.0, 128.9, 127.0, 115.7, 103.4, 61.9, 60.4, 44.4, 31.0, 29.7, 29.4, 26.6, 26.1, 25.6, 25.1, 24.0, 22.7, 21.1, 14.2, 14.1; HRMS (EI+) calcd for C_20_H_26_N_2_O_2_ [M]^+^: 326.1994, found: 326.1996.

#### 3.2.16. 4-Allyl-*N*-benzyl-2-phenyl-4,5-dihydrooxazole-4-carboxamide (**3j**)

Yield 84%, a colorless sticky oil; ^1^H NMR (300 MHz, CDCl_3_) δ 8.56 (s, 1H), 7.80 (d, *J* = 7.1 Hz, 2H), 7.50–7.24 (m, 8H), 6.01 (s, 1H), 5.88–5.79 (m, 1H), 5.29–5.19 (m, 2H), 5.10 (s, 1H), 4.73 (s, 2H), 4.08 (s, 2H), 1.41–0.72 (m, 2H); ^13^C NMR (125 MHz, CDCl_3_) δ 168.5, 165.9, 136.4, 135.0, 133.9, 131.9, 128.8, 128.6, 127.5, 127.2, 118.3, 104.4; HRMS (EI+) calcd for C_20_H_20_N_2_O_2_ [M]^+^: 320.1525, found: 320.1527.

#### 3.2.17. *N*-benzyl-4-cinnamyl-2-phenyl-4,5-dihydrooxazole-4-carboxamide (**3k**)

Yield 70%, a colorless sticky oil; ^1^H NMR (500 MHz, CDCl_3_) δ 8.32 (s, 1H), 7.84–7.81 (m, 2H), 7.53–7.50 (m, 1H), 7.54–7.42 (m, 2H), 7.37–7.28 (m, 9H), 6.50 (d, *J* = 15.9 Hz, 1H), 6.19–6.18 (m, 2H), 5.20 (s, 1H), 4.9 (s, 1H), 4.25 (s, 1H); ^13^C NMR (125 MHz, CDCl_3_) δ 168.4, 166.0, 136.4, 136.3, 134.7, 134.1, 132.0, 128.9, 128.7, 128.7, 128.0, 127.7, 127.4, 127.2, 126.5, 104.5, 30.9, 34.4; HRMS (EI+) calcd for C_26_H_24_N_2_O_2_ [M]^+^: 396.1838, found: 396.1840.

#### 3.2.18. (*E*)-*N*-benzyl-4-(but-2-en-1-yl)-2-phenyl-4,5-dihydrooxazole-4-carboxamide (**3l**)

Yield 70%, colorless sticky oil; ^1^H NMR (500 MHz, CDCl_3_) δ 8.63 (s, 1H), 7.84–7.81 (m, 2H), 7.38–7.14 (m, 10H), 5.87(s, 1H), 5.50–5.35 (m, 2H), 4.94 (s,1H), 4.60 (s, 2H), 3.90 (s, 2H); ^13^C NMR (125 MHz, CDCl_3_) δ 168.4, 166.0, 137.1, 136.7, 134.8, 134.1, 134.0, 131.9, 128.8, 128.7, 128.6, 127.4, 127.1, 104.3, 60.4, 30.3, 25.3, 21.1, 14.2, 13.4; HRMS (EI+) calcd for C_21_H_22_N_2_O_2_ [M]^+^: 334.1682, found: 334.1681.

#### 3.2.19. (*E*)-*N*-benzyl-4-(pent-2-en-1-yl)-2-phenyl-4,5-dihydrooxazole-4-carboxamide (**3m**)

Yield 74%, colorless oil; ^1^H NMR (500 MHz, CDCl_3_) δ 8.35 (s, 1H), 7.83–7.82 (t, *J* = 7.2 Hz, 2H), 7.54–7.50 (m, 1H), 7.46–7.43 (m, 2H), 7.37–7.35 (t, *J* = 7.2 Hz, 2H), 7.30–7.26 (m, 3H), 6.17 (s, 1H), 5.65–5.59 (m, 1H), 5.45-5.40 (m, 1H), 5.13 (s, 1H), 4.73 (s, 2H), 4.13–4.10 (m, 2H), 1.94–1.89 (m, 2H), 0.93–0.90 (m, 3H); ^13^C NMR (125 MHz, CDCl_3_) δ 168.3, 166.0, 137.1, 136.7, 134.8., 134.1, 131.9, 128.8, 128.6, 127.5, 127.2, 104.4, 60.4, 30.9, 21.1, 14.2, 13.4; HRMS (EI+) calcd for C_22_H_24_N_2_O_2_ [M]^+^: 348.1838, found: 348.1841.

#### 3.2.20. (*Z*)-*N*-benzyl-4-(pent-2-en-1-yl)-2-phenyl-4,5-dihydrooxazole-4-carboxamide (**3n**)

Yield 75%, colorless sticky oil; ^1^H NMR (500 MHz, CDCl_3_) δ 8.49 (s, 1H), 7.82 (d, *J* = 5.0 Hz, 2H), 7.51–7.50 (m, 1H), 7.49–7.48 (m, 2H), 7.34–7.40 (m, 2H), 7.28–7.25 (m, 3H), 6.12 (s, 1H), 5.64–5.60 (m, 1H) 5.45–5.42 (m, 1H), 5.14 (s, 1H), 4.71 (s, 2H), 4.03 (s, 2H) 2.06 (t, *J* = 7.0 Hz), 0.98 (t, *J* = 7.5 Hz); ^13^C NMR (125 MHz, CDCl_3_) δ 168.3, 166.0, 137.1, 136.7, 134.8, 134.1, 131.9, 128.8, 128.6, 127.5, 127.2, 104.4, 60.4, 30.9, 25.3, 21.1, 14.2, 13.4; HRMS (EI+) calcd for C_22_H_24_N_2_O_2_ [M]^+^: 348.1838, found: 348.1836.

## 4. Conclusions

The unexpected rearrangement of *N*-allyl-2-phenyl-4,5-dihydrooxazole-4-carboxamides in the presence of LiHMDS was discovered. Several key features were noted: (1) The net reaction consisted of 1,3-migration of the *N*-allyl group. (2) The rearrangement produced a congested aza-quaternary carbon center. (3) Both cyclic and acyclic substrates underwent the unexpected rearrangement to afford products in moderate to high yields. (4) The reaction seemed to be highly stereoselective. Although the reaction mechanism has not yet been confirmed, the method might be useful for the synthesis of challenging targets that possess aza-quaternary carbon centers. These centers are found in a variety of bioactive alkaloids and small molecules. 

## Figures and Tables

**Table 1:** Optimization of the Reaction ^a^.

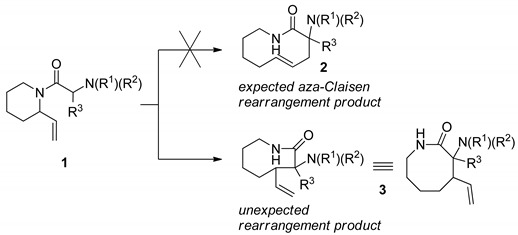					
Entry	Substrate	Conditions	Time (h)	Yield 2 (%)	Yield 3 (%) ^b^
**1**	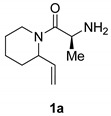	LiHMDS toluene reflux	4	-	ND ^c^
**2**	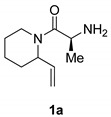	*i*PrMgCl benzene reflux	4	-	ND ^c^
**3**	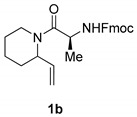	LiHMDS toluene reflux	4	-	ND ^c^
**4**	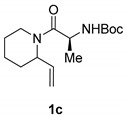	LiHMDS toluene reflux	4	-	ND ^c^
**5**	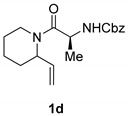	LiHMDS toluene reflux	4	-	ND ^c^
**6**	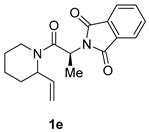	LiHMDS toluene reflux	4	-	ND ^c^
**7**	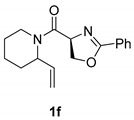	LiHMDS toluene reflux	1	-	72 ^d^
**8**	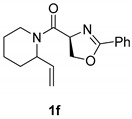	LiHMDS toluene rt	1	-	92 ^d^
**9**	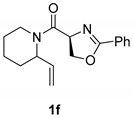	LiHMDS benzene rt	1	-	96 ^d^
**10**	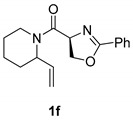	*i*PrMgCl benzene rt	1	-	84 ^d^

^a^ Reaction conditions: Three equivalents of either lithium bis(trimethylsilyl)amide (LiHMDS) or isopropylmagnesium chloride (*i*PrMgCl) were added dropwise to the given substrate in solution and either refluxed or reacted at room temperature (rt). ^b^ Isolated yields. ^c^ Not detected. ^d^ Single diastereomer, not determined.

**Table 2:** Scope of the reaction ^a^.

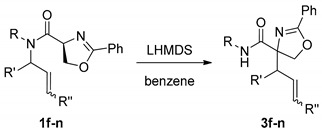				
Entry	Substrate	Product	Time	Yield (%) ^b^
**1**	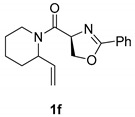	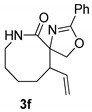	1 h	96 ^c^
**2**	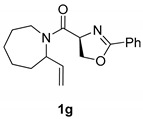	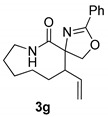	1 h	71 ^c^
**3**	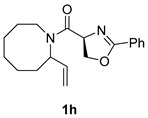	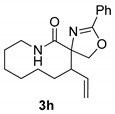	1 h	76 ^c^
**4**	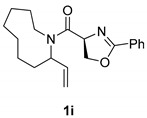	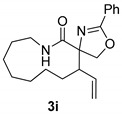	1 h	77 ^c^
**5**	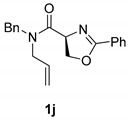	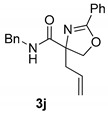	5 min	95
**6**	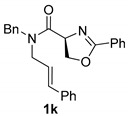	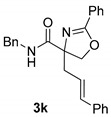	12 h ^d^	70
**7**	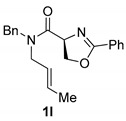	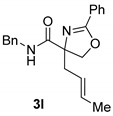	12 h ^d^	84
**8**	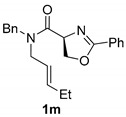	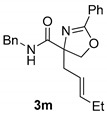	12 h ^d^	75
**9**	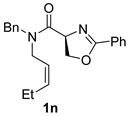	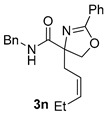	12 h ^d^	74

^a^ Reactions were performed with three equivalents of LiHMDS, at room temperature unless otherwise noted. ^b^ Isolated yields. ^c^ Single diastereomer, not determined. ^d^ Refluxed.

## Supplementary Materials

The following are available online. ^1^H and ^13^C NMR spectra of **1f-n** and **3f-n**.

## References

[1] Martín Castro A.M. (2004). Claisen rearrangement over the past nine decades. Chem. Rev..

[2] Jung J.W., Kim S.H., Suh Y.G. (2017). Advances in Aza-Claisen-Rearrangement-Induced Ring-Expansion Strategies. Asian J. Org. Chem..

[3] Nowicki J. (2000). Claisen, Cope and related rearrangements in the synthesis of flavour and fragrance compounds. Molecules.

[4] Kazmaier U. (1996). Application of the ester enolate Claisen rearrangement in the synthesis of amino acids containing quaternary carbon centers. J. Org. Chem..

[5] Curran D.P., Suh Y.G. (1984). Substituent effects on the Claisen rearrangement. The accelerating effect of a 6-donor substituent. J. Am. Chem. Soc..

[6] Aviyente V., Yoo H.Y., Houk K. (1997). Analysis of substituent effects on the Claisen rearrangement with Ab Initio and density functional theory. J. Org. Chem..

[7] Aviyente V., Houk K. (2001). Cyano, amino, and trifluoromethyl substituent effects on the Claisen rearrangement. J. Phys. Chem. A.

[8] Suh Y.-G., Lee Y.-S., Kim S.-H., Jung J.-K., Yun H., Jang J., Kim N.-J., Jung J.-W. (2012). A stereo-controlled access to functionalized macrolactams via an aza-Claisen rearrangement. Org. Biomol. Chem.

[9] Majumdar K., Bhattacharyya T., Chattopadhyay B., Sinha B. (2009). Recent advances in the aza-Claisen rearrangement. Synthesis.

[10] Zahedi E., Ali-Asgari S., Keley V. (2010). NBO and NICS analysis of the allylic rearrangements (the Cope and 3-aza-Cope rearrangements) of hexa-1, 5-diene and *N*-vinylprop-2-en-1-amine: A DFT study. Cent. Eur. J. Chem..

[11] Tsunoda T., Tatsuki S., Shiraishi Y., Akasaka M., Itô S. (1993). Asymmetric aza-Claisen rearrangement of glycolamide and glycinamide enolates. Synthesis of optically active α-hydroxy and α-amino acids. Tetrahedron Lett..

[12] Yoshizuka M., Nishii T., Sasaki H., Kitakado J., Ishigaki N., Okugawa S., Kaku H., Horikawa M., Inai M., Tsunoda T. (2011). Promotion of asymmetric aza-Claisen rearrangement of *N*-allylic carboxamides using excess base. Synlett.

[13] Jung J.-K., Choi N.-S., Suh Y.-G. (2004). Functional divergency oriented synthesis of azoninones as the key intermediates for bioactive indolizidine alkaloids analogs. Arch. Pharm. Res..

[14] Jang J., Jung J.-W., Ahn J., Sim J., Chang D.-J., Kim D.-D., Suh Y.-G. (2012). Asymmetric formal synthesis of schulzeines A and C. Org. Biomol. Chem.

[15] Kim S.-H., Lee W.-I., Kim S.-M., Jung J.-K., Jang J., Sim J., Jung J.-W., Suh Y.-G. (2016). Studies on the aza-Claisen rearrangement of 7 to 9-membered vinylazacycles. Heterocycles.

[16] Clayden J., Donnard M., Lefranc J., Tetlow D.J. (2011). Quaternary centres bearing nitrogen (α-tertiary amines) as products of molecular rearrangements. Chem. Commun..

[17] Kim M., Jang J., Choi G., Chung S., Lim C., Hur J., Kim H., Na Y., Son W., Suh Y.-G. (2018). Conversion of Medium-Sized Lactams to α-Vinyl or α-Acetylenyl Azacycles via *N, O*-Acetal TMS Ethers. Molecules.

[18] Denoël T., Zervosen A., Lemaire C., Plenevaux A., Luxen A. (2014). Synthesis of protected α-alkyl lanthionine derivatives. Tetrahedron.

[19] Ghigo G., Cagnina S., Maranzana A., Tonachini G. (2010). The mechanism of the Stevens and Sommelet− Hauser rearrangements. A theoretical study. J. Org. Chem..

[20] Bott T.M., Vanecko J.A., West F. (2009). One-carbon ring expansion of azetidines via ammonium ylide [1,2]-shifts: A simple route to substituted pyrrolidines. J. Org. Chem..

[21] Soheili A., Tambar U.K. (2011). Tandem Catalytic Allylic Amination and [2,3]-Stevens Rearrangement of Tertiary Amines. J. Am. Chem. Soc..

[22] Titov A., Samavati R., Alexandrova E., Borisova T., Dang Thi T., Nguyen V., Le T., Varlamov A., Van der Eycken E., Voskressensky L. (2018). Synthesis of 1-(para-methoxyphenyl) tetrazolyl-Substituted 1, 2, 3, 4-Tetrahydroisoquinolines and Their Transformations Involving Activated Alkynes. Molecules.

