# Supplementary material for: Unexpected Rearrangement of N-Allyl-2-phenyl-4,5-Dihydrooxazole-4-Carboxamides to Construct Aza-Quaternary Carbon Centers
Source: Molecules. 2019 Dec 8;24(24):4495. doi: 10.3390/molecules24244495 (PMC6943665; doi:10.3390/molecules24244495)

Supplementary Materials

Unexpected Rearrangement of *N*-Allyl-2-phenyl-4,5-dihydrooxazole-4-carboxamides to Construct Aza-Quaternary Carbon Centers

Goyeong Choi,[1] Seoyoung Jo,[1] Juyeon Mun,[1] Yonguk Jeong,[1] Seok-Ho Kim[2] and Jong-Wha Jung[1,*]

[1] College of Pharmacy, Research Institute of Pharmaceutical Sciences, Kyungpook National University, Daegu 41566, Republic of Korea

[2] College of Pharmacy, CHA University, Gyeonggi-do 11160, Republic of Korea

* Correspondence: jungj@knu.ac.kr; Tel.: +82-53-950-8578 (J.-W.J.)

^{1}H and ^{13}C NMR Spectra

(2-Phenyl-4,5-dihydrooxazol-4-yl)(2-vinylpiperidin-1-yl)methanone (**1f**)

^{1}H-NMR

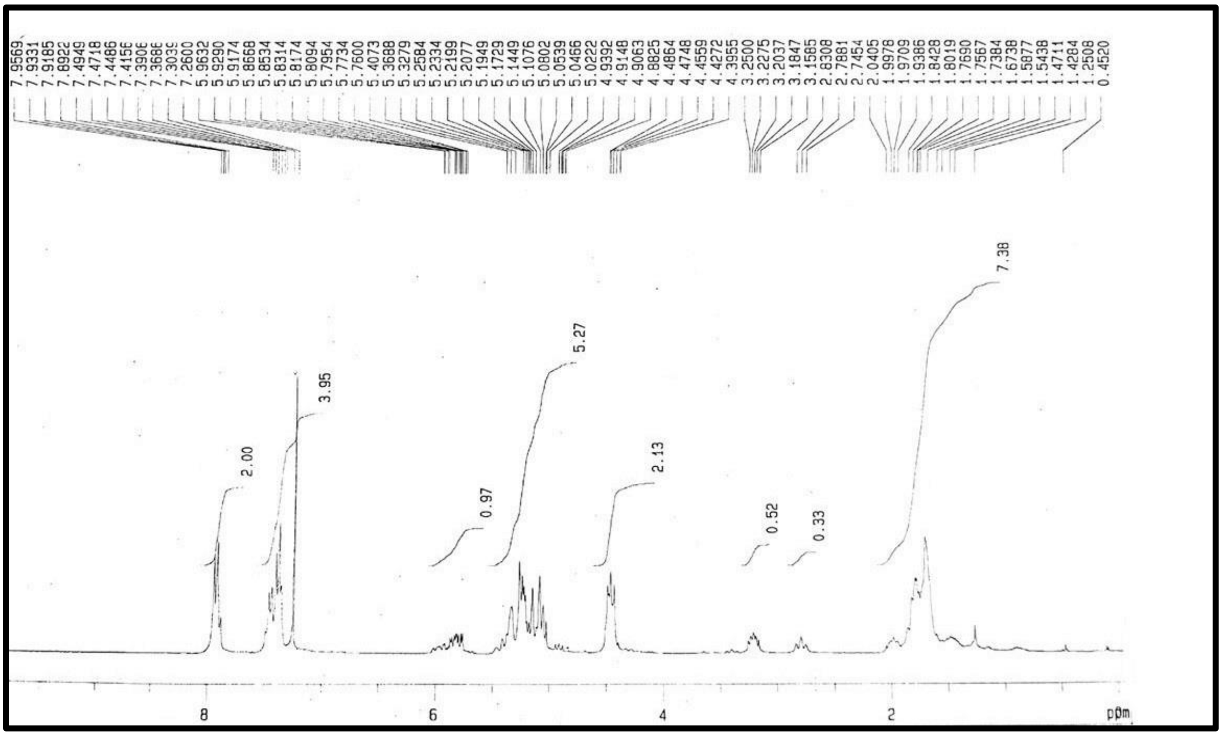

^{13}C-NMR

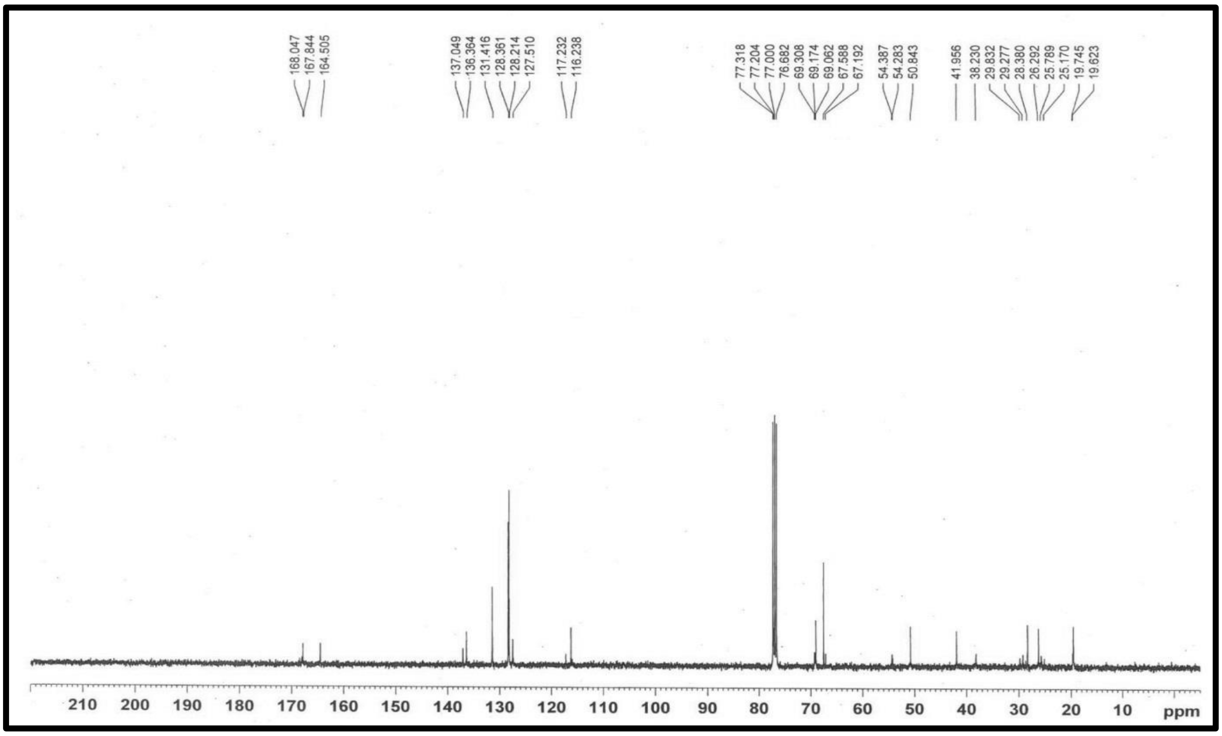

(2-Phenyl-4,5-dihydrooxazol-4-yl)(2-vinylazepan-1-yl)methanone (**1g**)

^{1}H-NMR

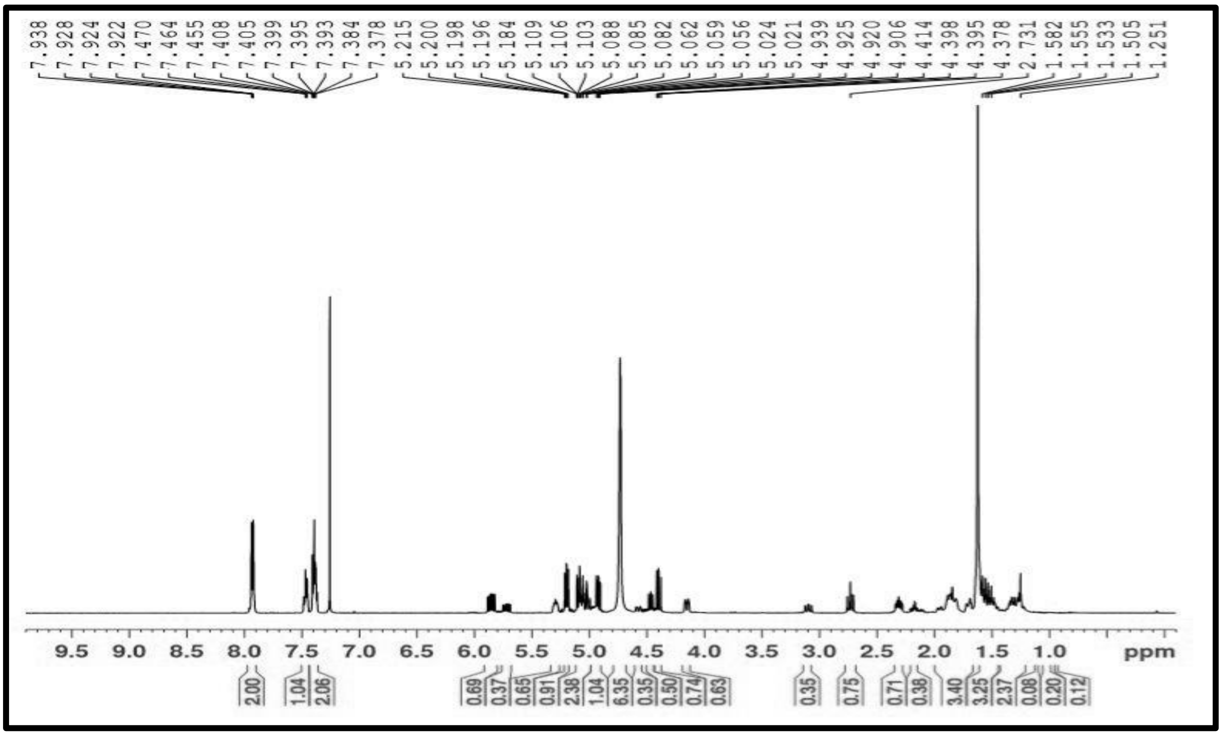

^{13}C-NMR

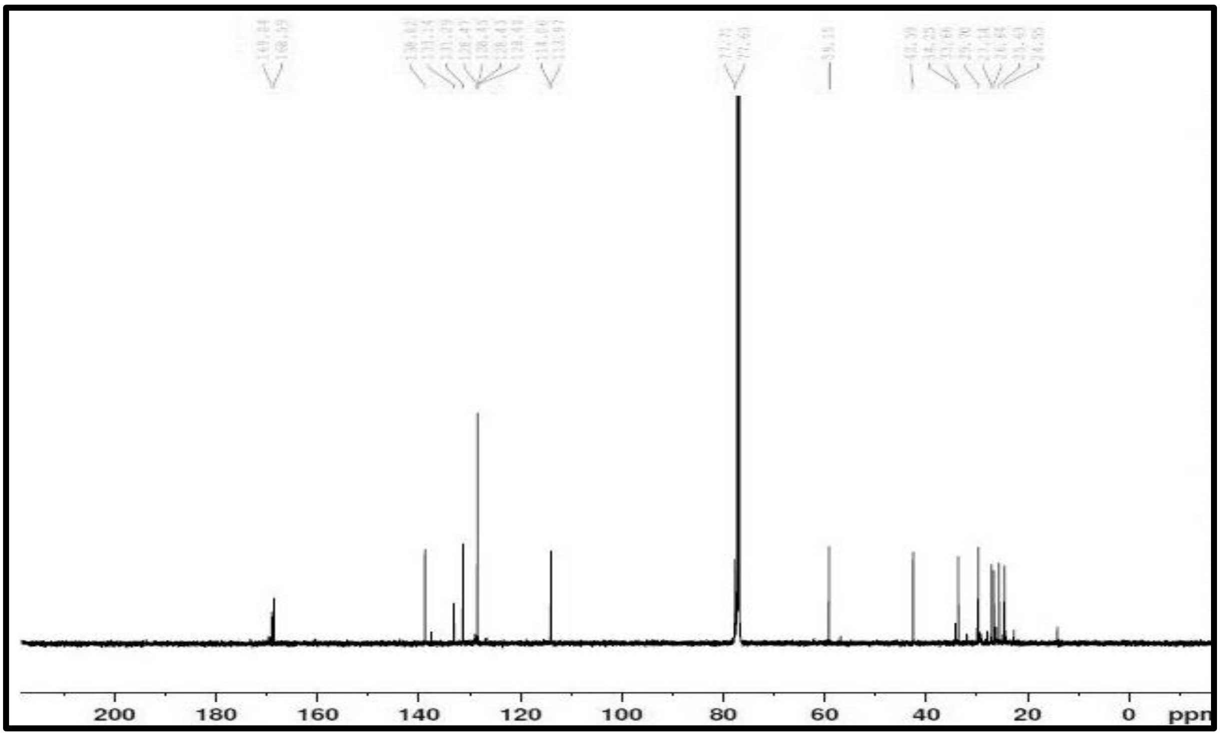

(2-Phenyl-4,5-dihydrooxazol-4-yl)(2-vinylazocan-1-yl)methanone (**1h**)

^{1}H-NMR

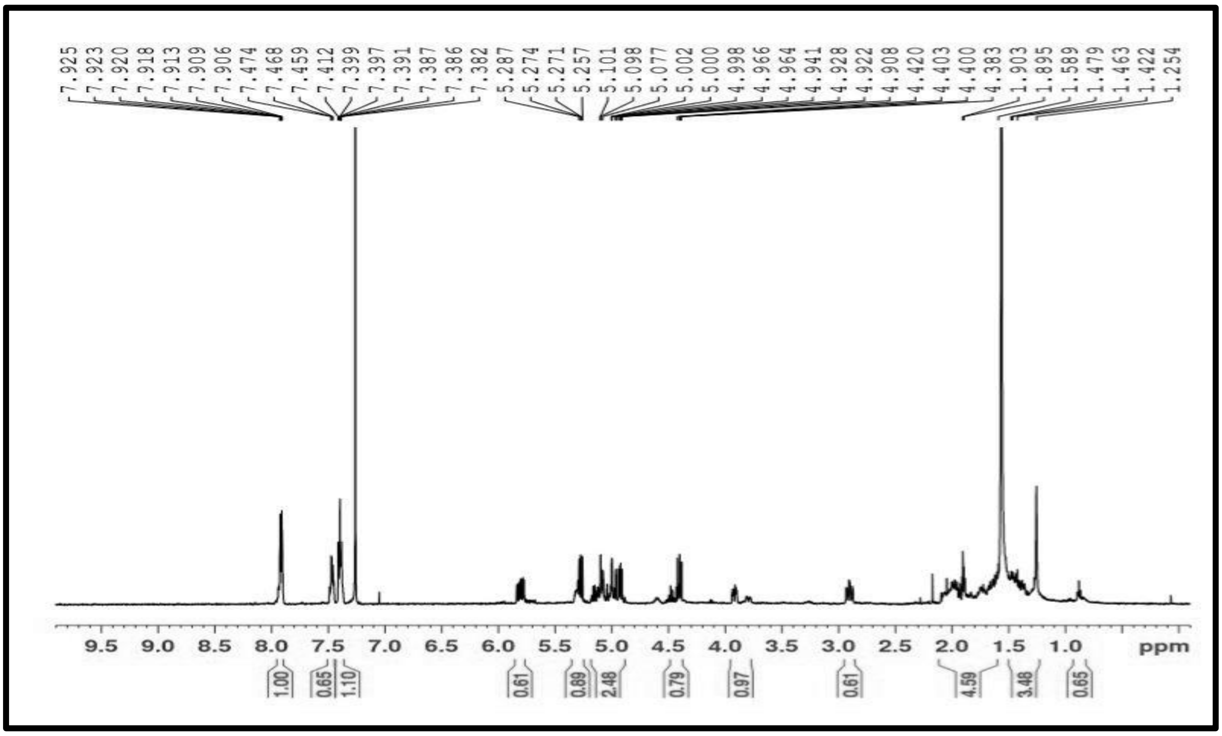

^{13}C-NMR

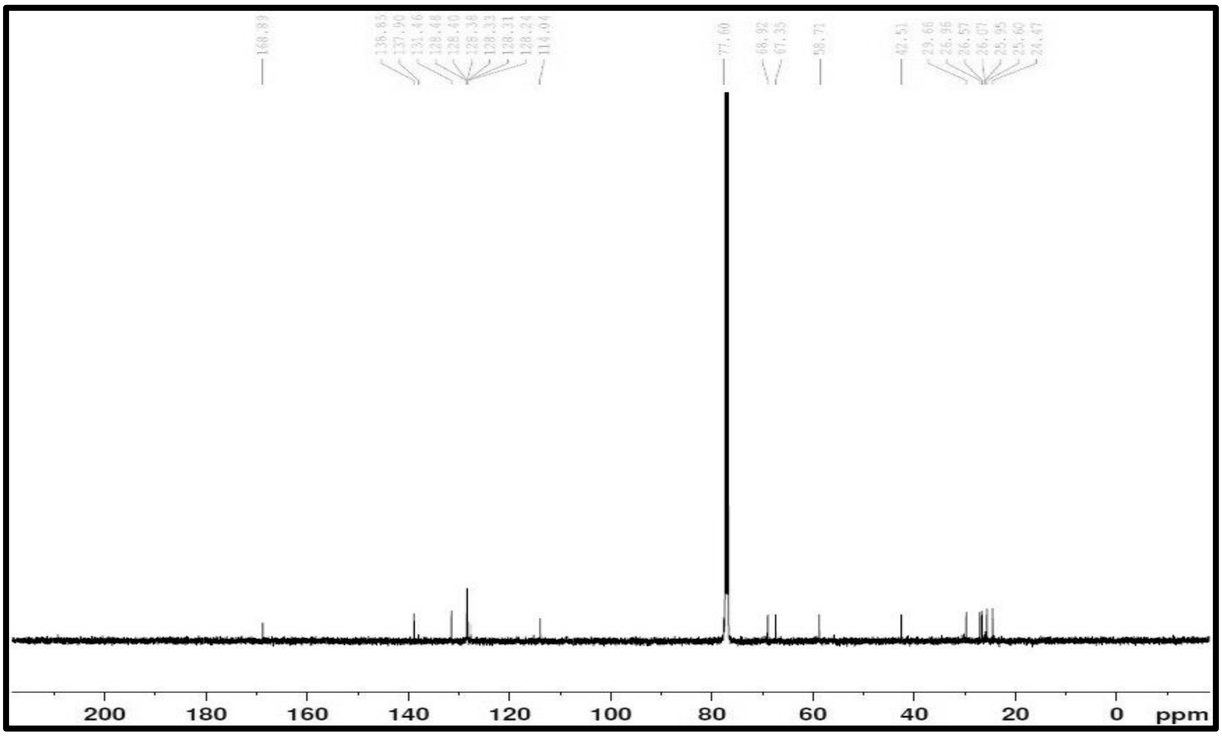

(2-Phenyl-4,5-dihydrooxazol-4-yl)(2-vinylazonan-1-yl)methanone (**1i**)

^{1}H-NMR

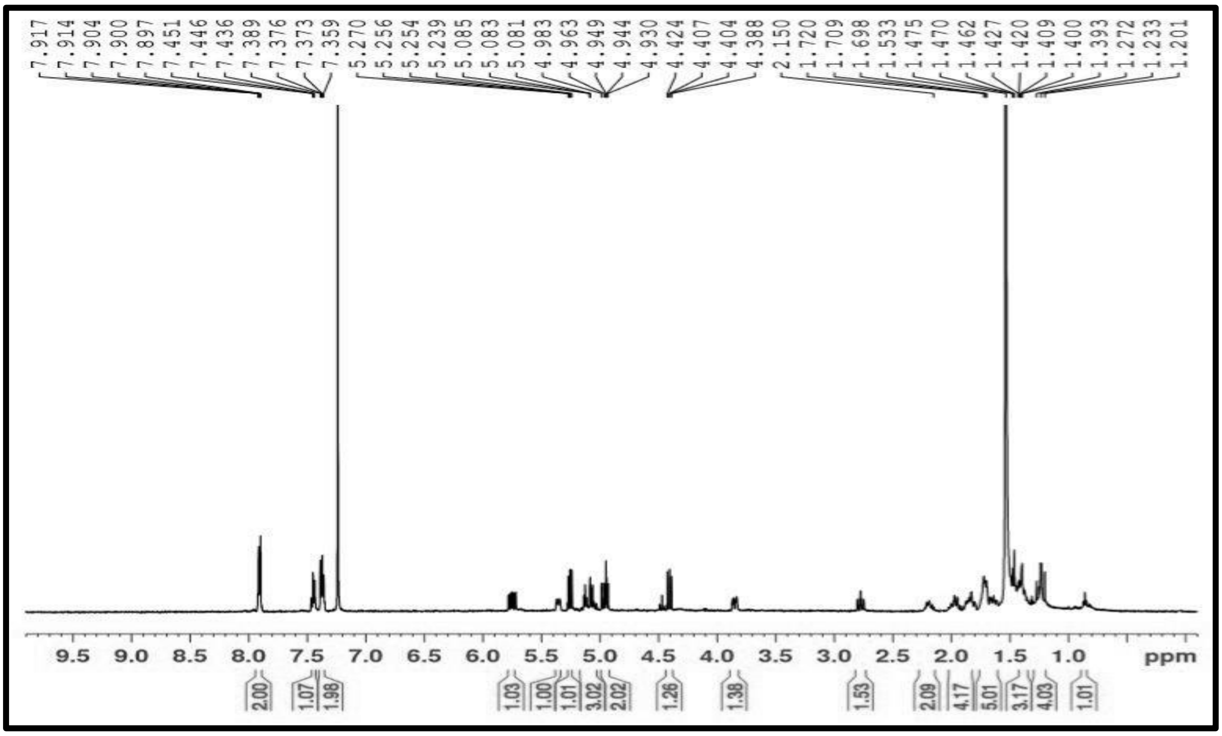

^{13}C-NMR

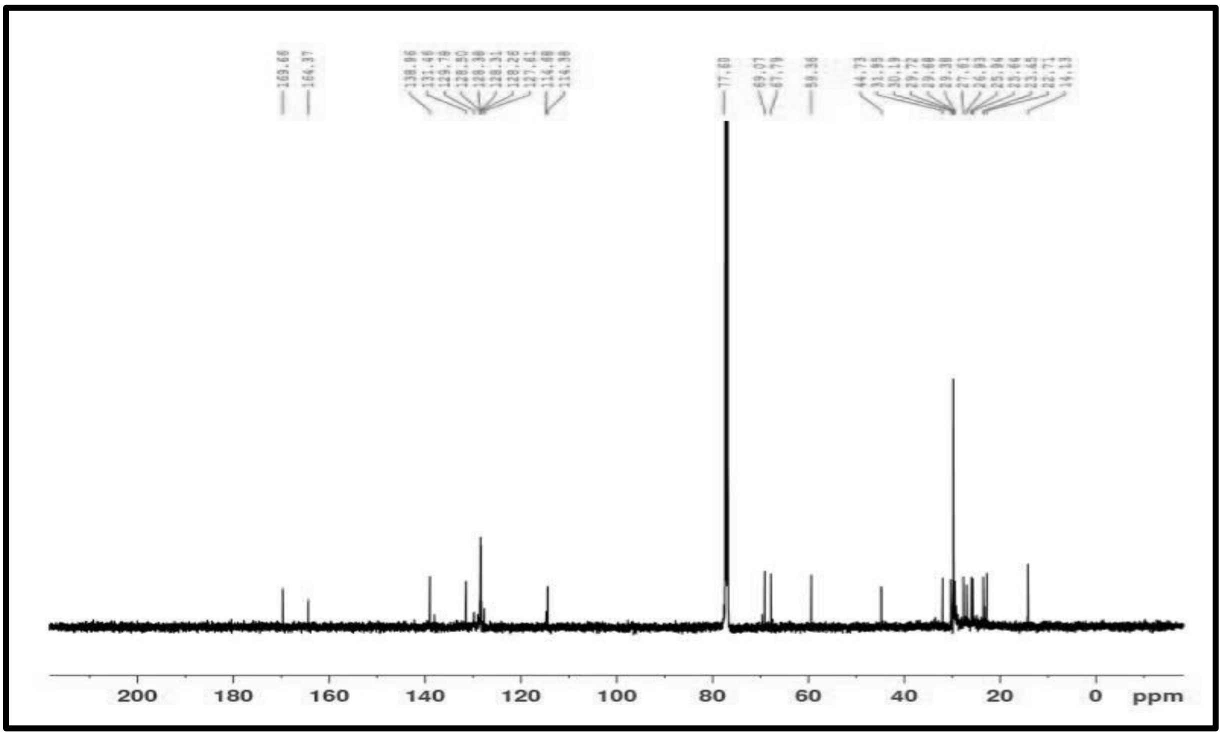

N-allyl-*N*-benzyl-2-phenyl-4,5-dihydrooxazole-4-carboxamide (**1j**)

^{1}H-NMR

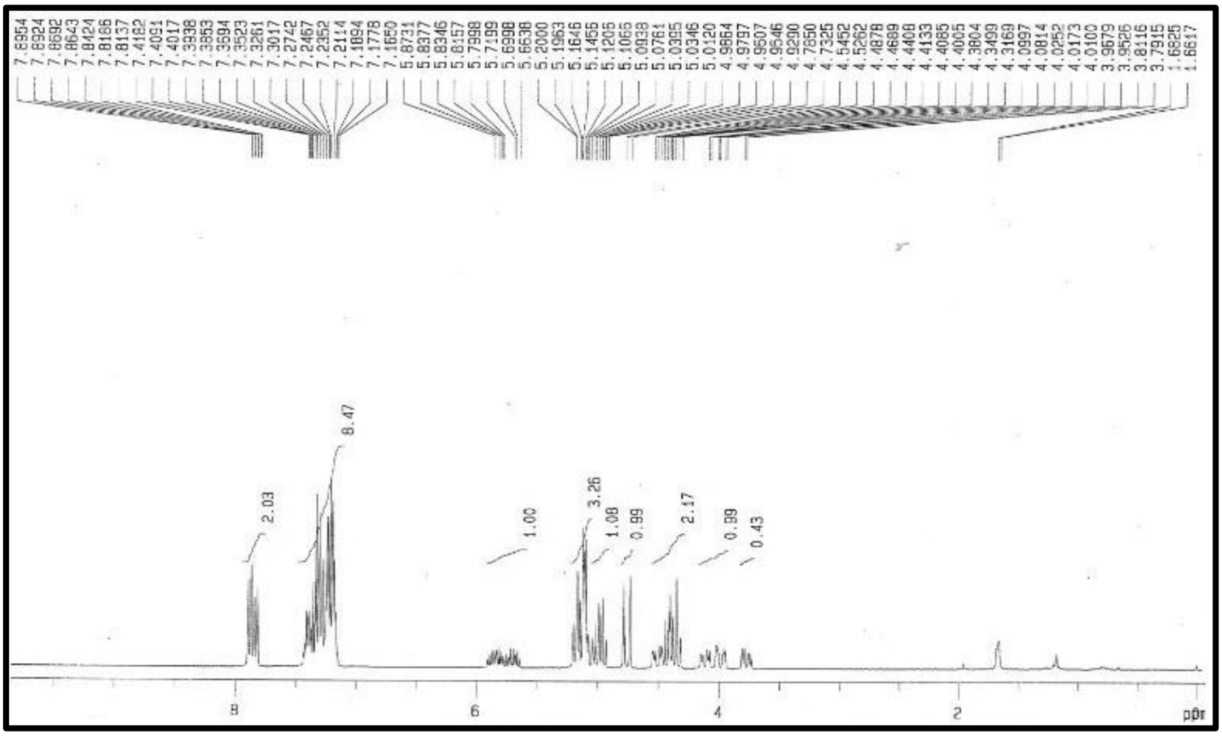

^{13}C-NMR

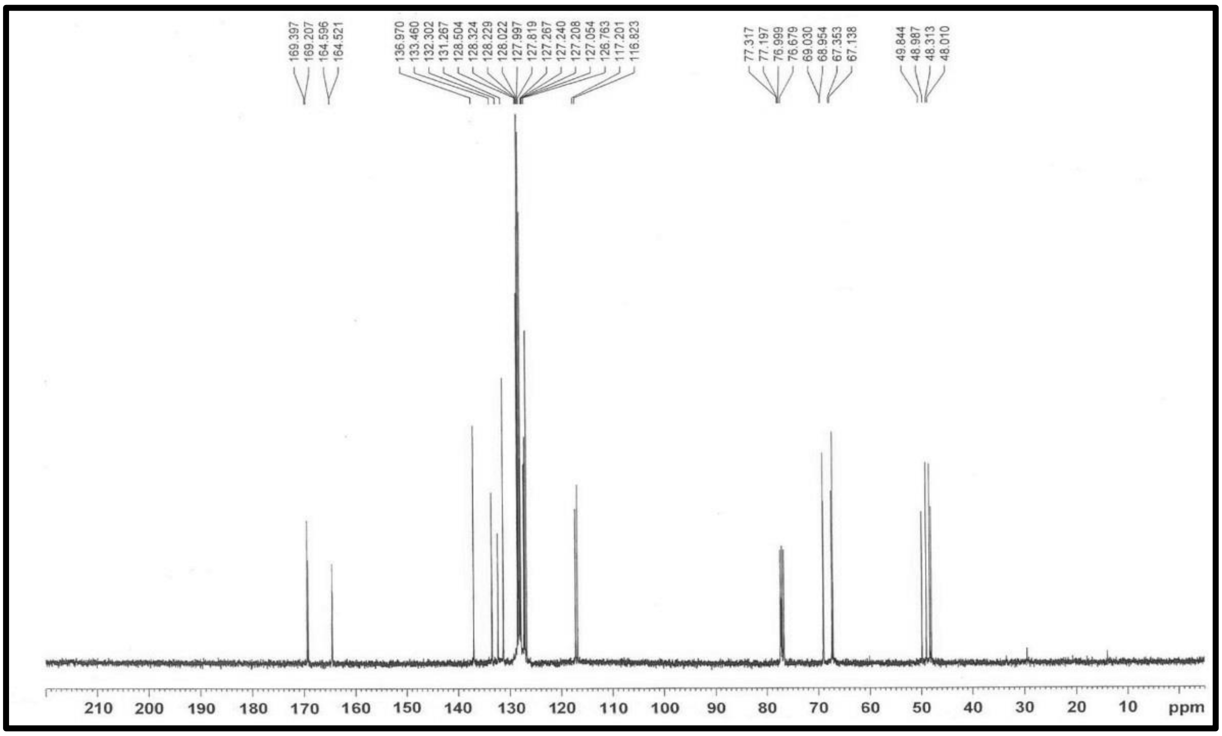

(E)-N-benzyl-N-cinnamyl-2-phenyl-4,5-dihydrooxazole-4-carboxamide (***1k***)

^{1}H-NMR

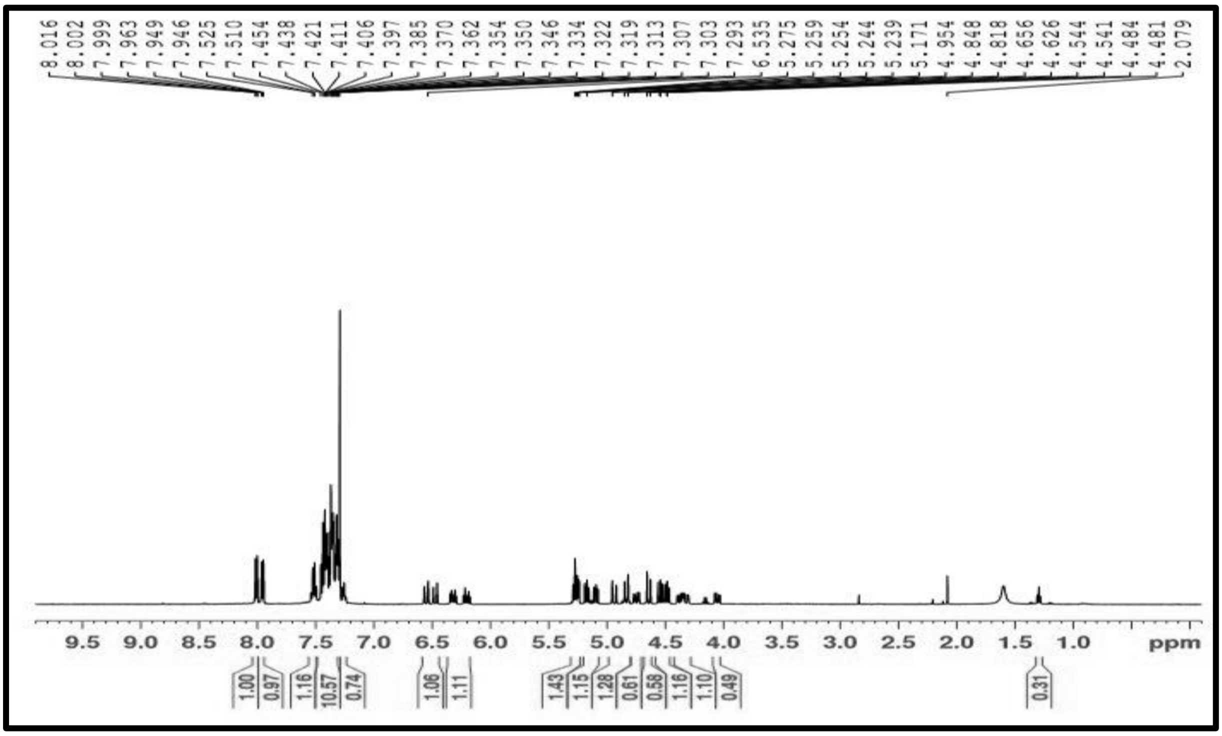

^{13}C-NMR

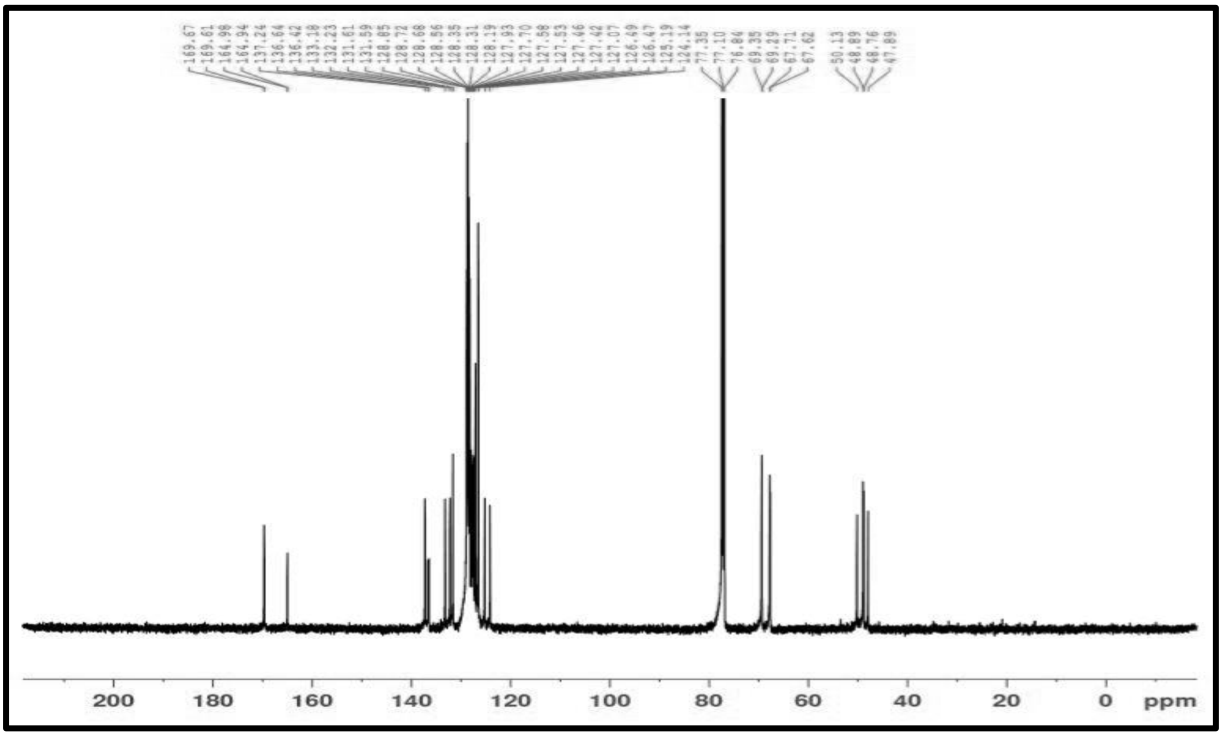

(*E*)-*N*-benzyl-*N*-(but-2-en-1-yl)-2-phenyl-4,5-dihydrooxazole-4-carboxamide (**1l**)

^{1}H-NMR

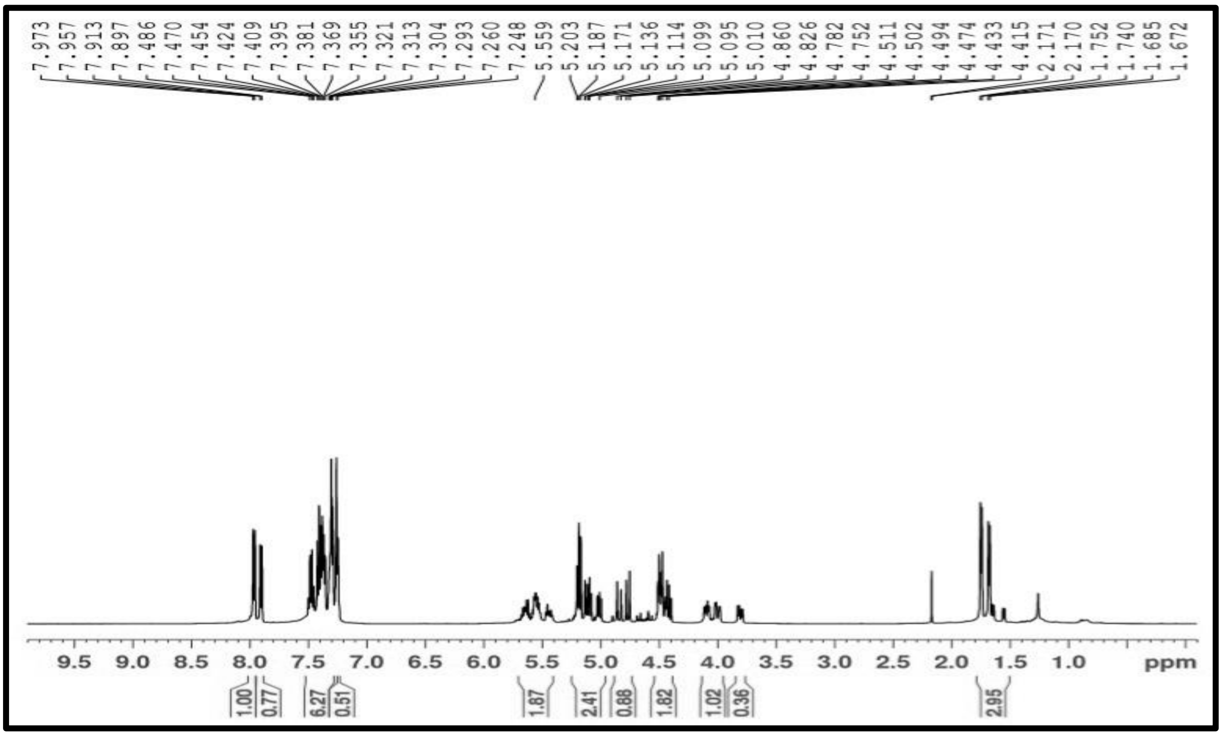

^{13}C-NMR

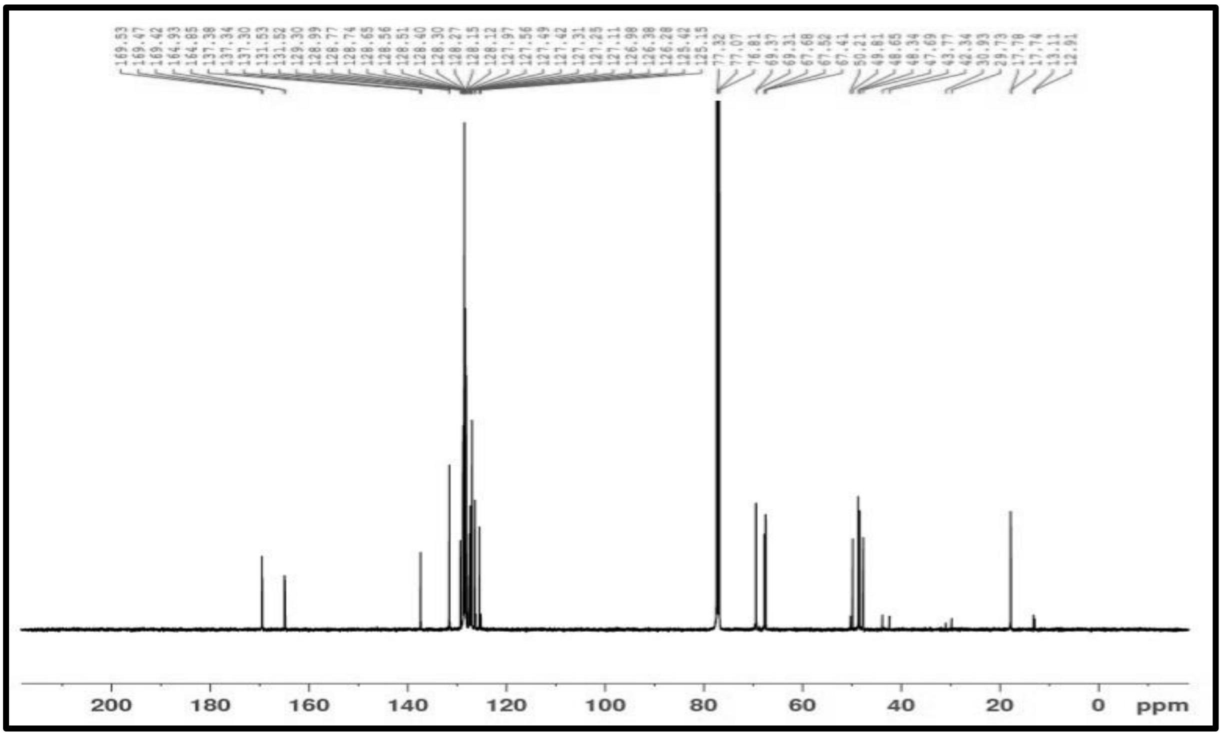

(*E*)-*N*-benzyl-*N*-(pent-2-en-1-yl)-2-phenyl-4,5-dihydrooxazole-4-carboxamide (**1m**)

^{1}H-NMR

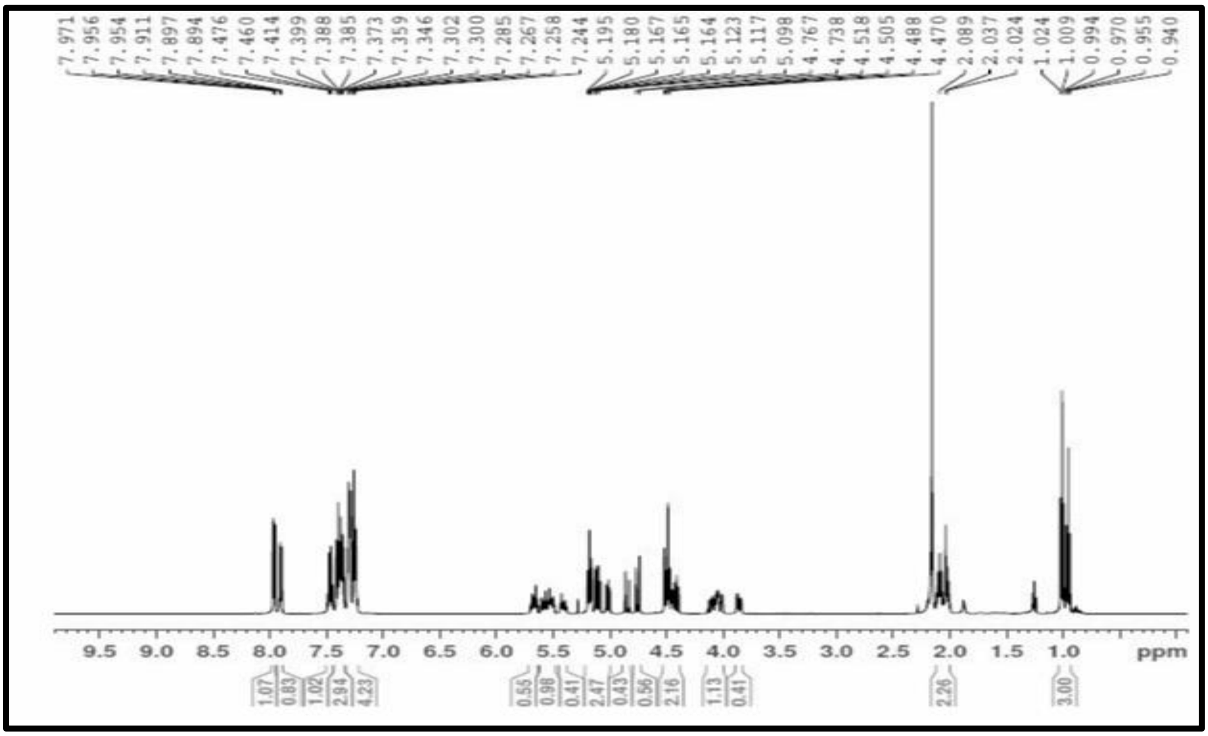

^{13}C-NMR

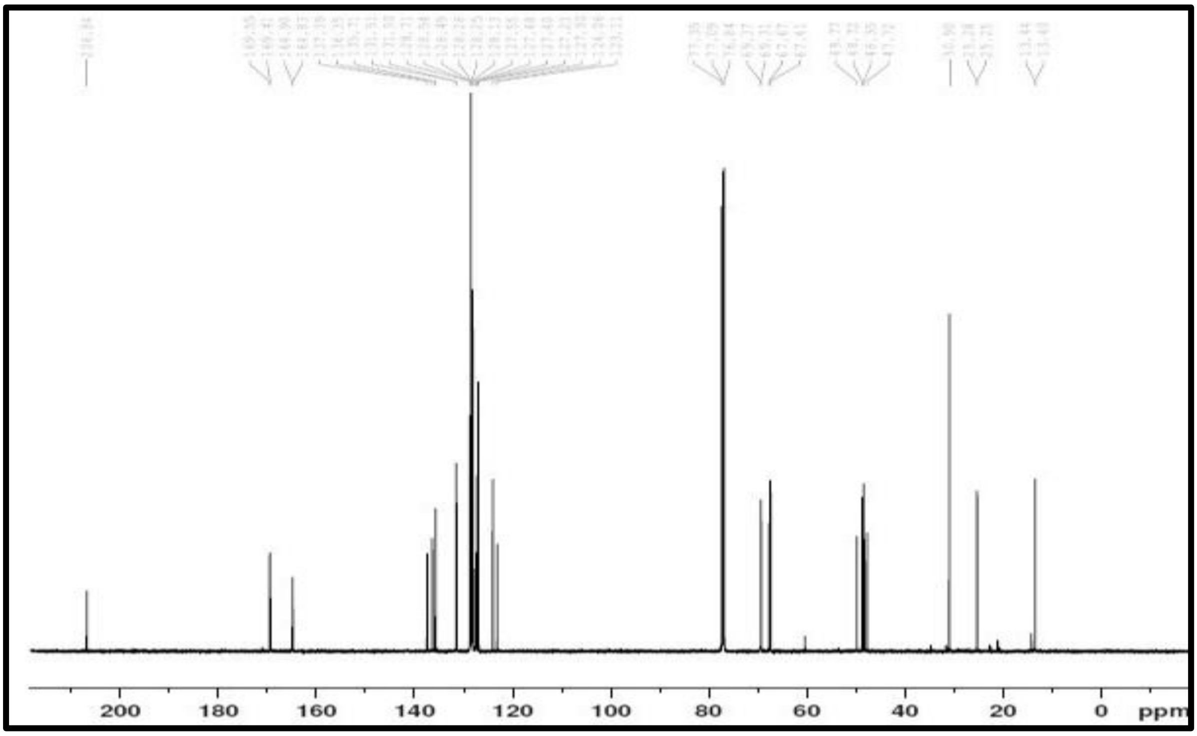

.

(*Z*)-*N*-benzyl-*N*-(pent-2-en-1-yl)-2-phenyl-4,5-dihydrooxazole-4-carboxamide (**1n**)

^{1}H-NMR

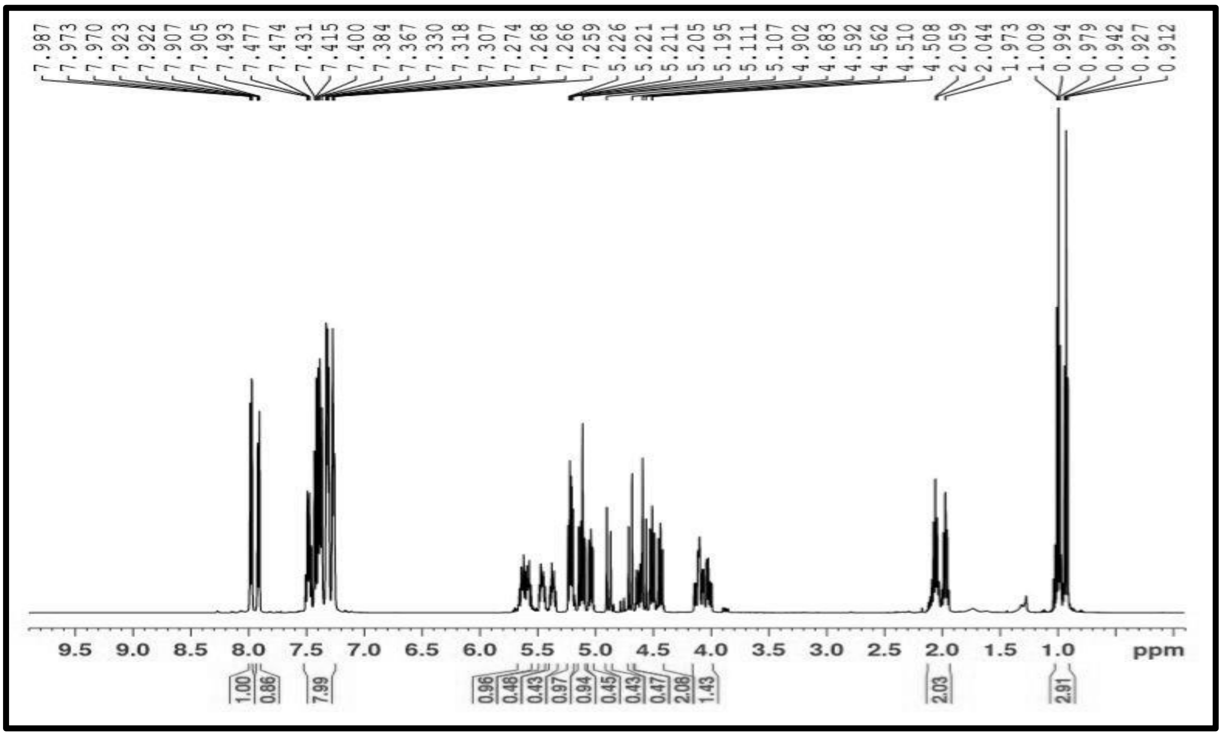

^{13}C-NMR

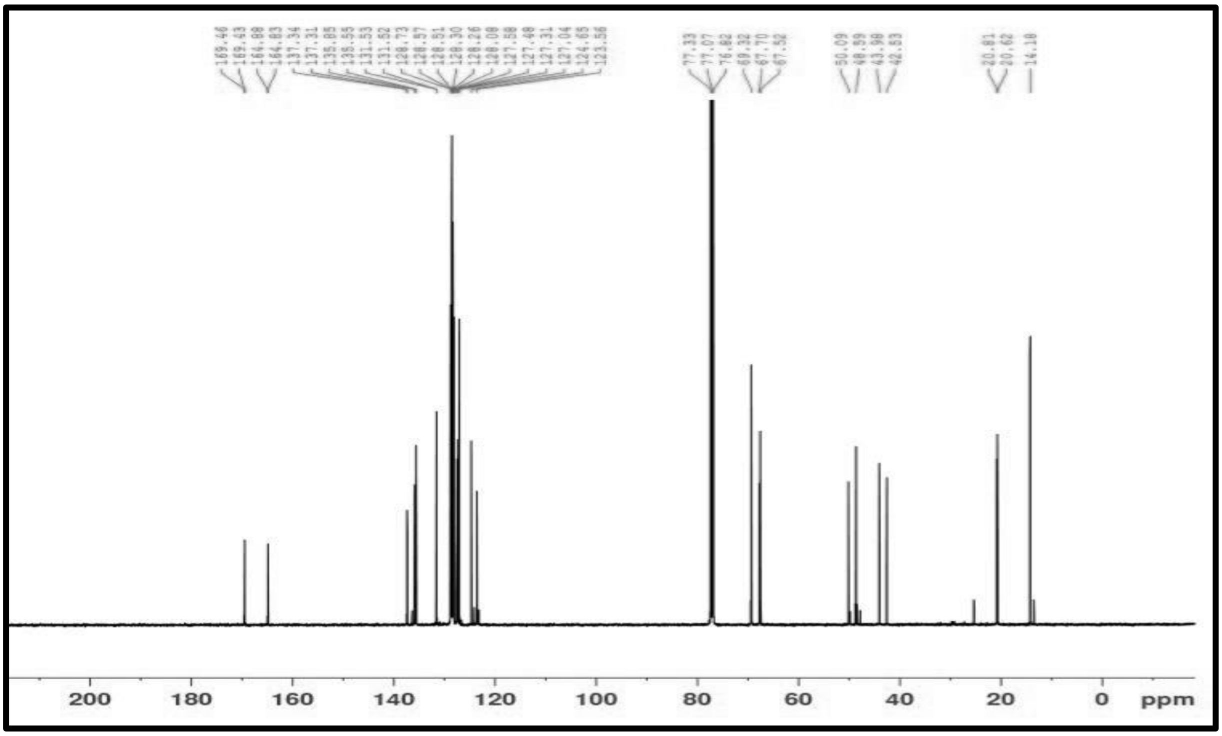

2-Phenyl-12-vinyl-3-oxa-1,7-diazaspiro[4.7]dodec-1-en-6-one (**3f**)

^{1}H-NMR

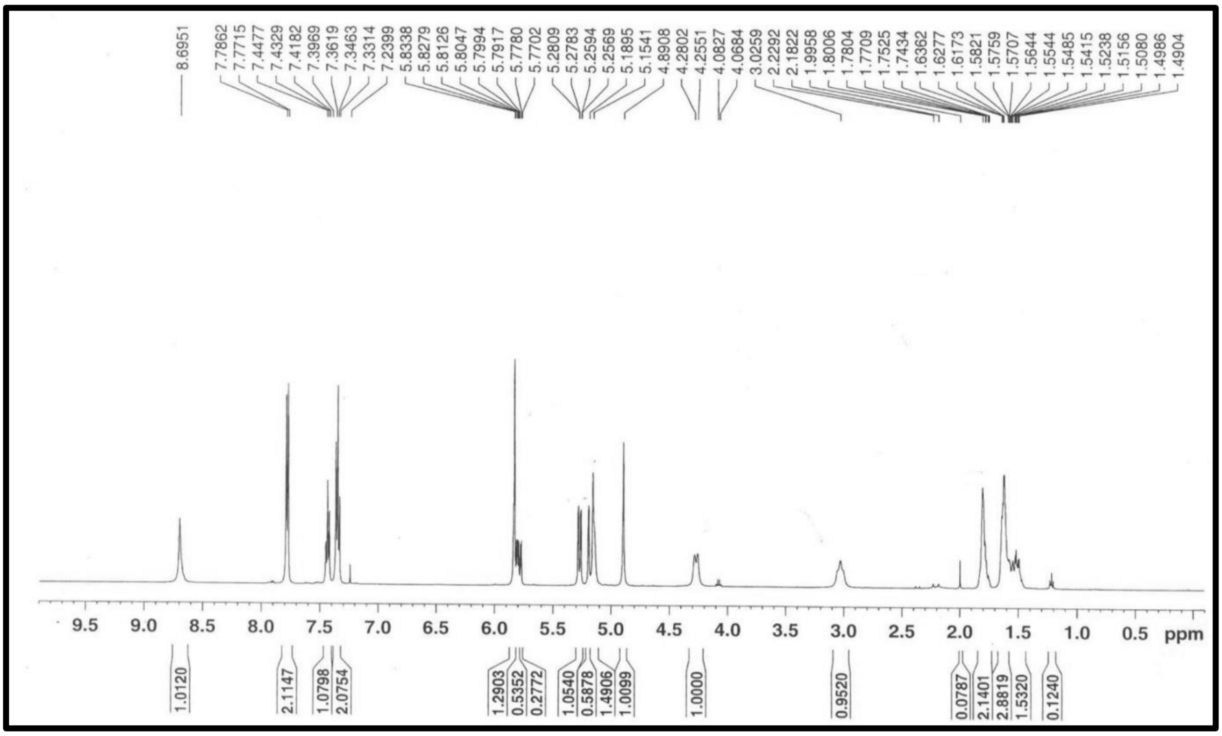

^{13}C-NMR

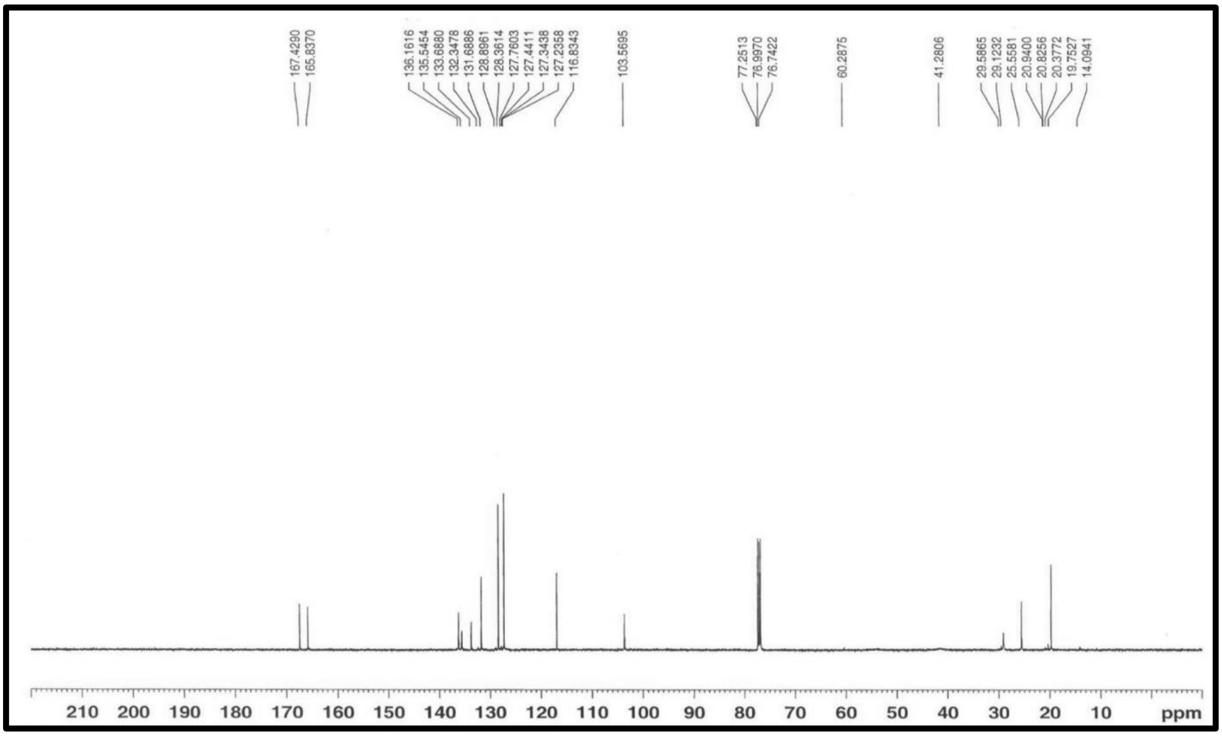

2-Phenyl-13-vinyl-3-oxa-1,7-diazaspiro[4.8]tridec-1-en-6-one (**3g**)

^{1}H-NMR

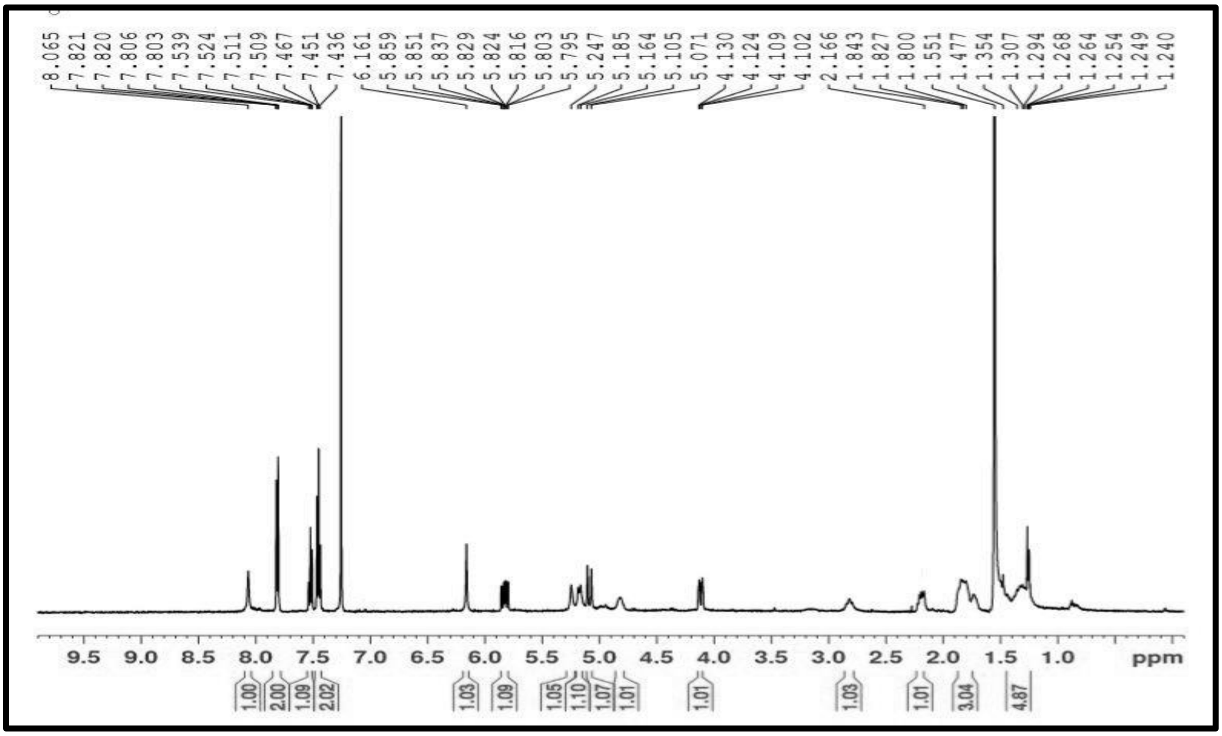

^{13}C-NMR

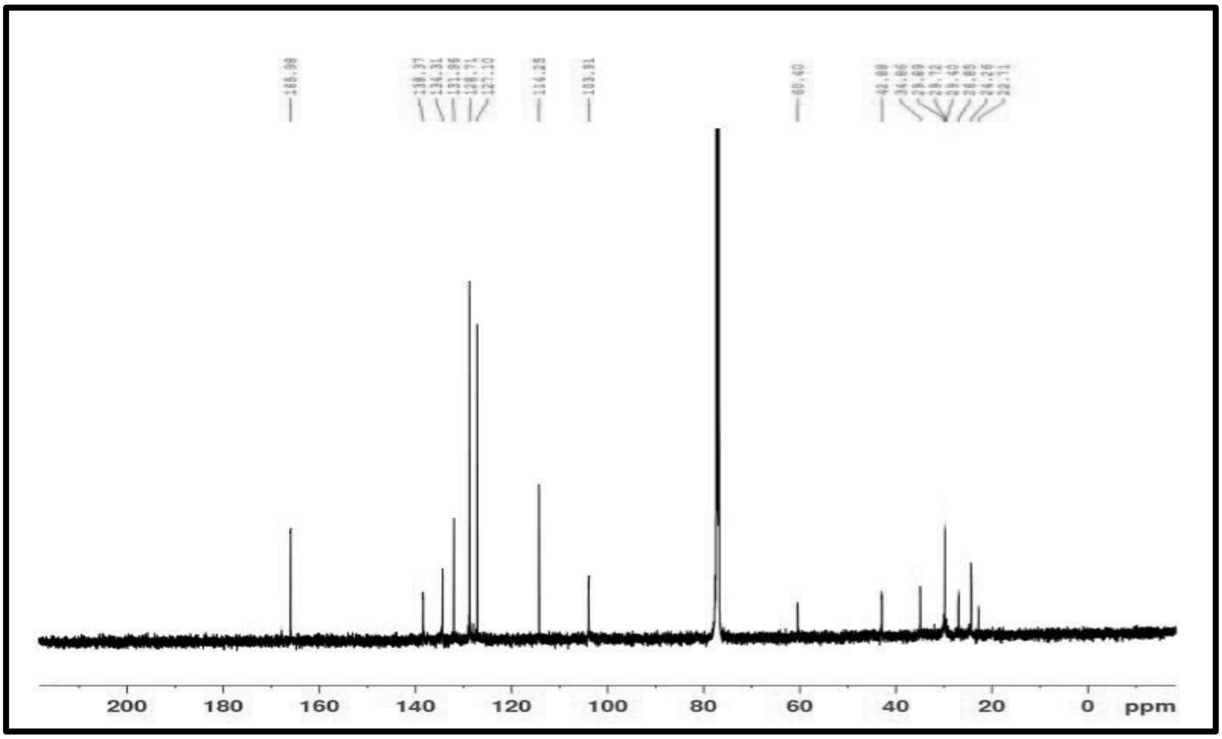

2-Phenyl-14-vinyl-3-oxa-1,7-diazaspiro[4.9]tetradec-1-en-6-one (**3h**)

^{1}H-NMR

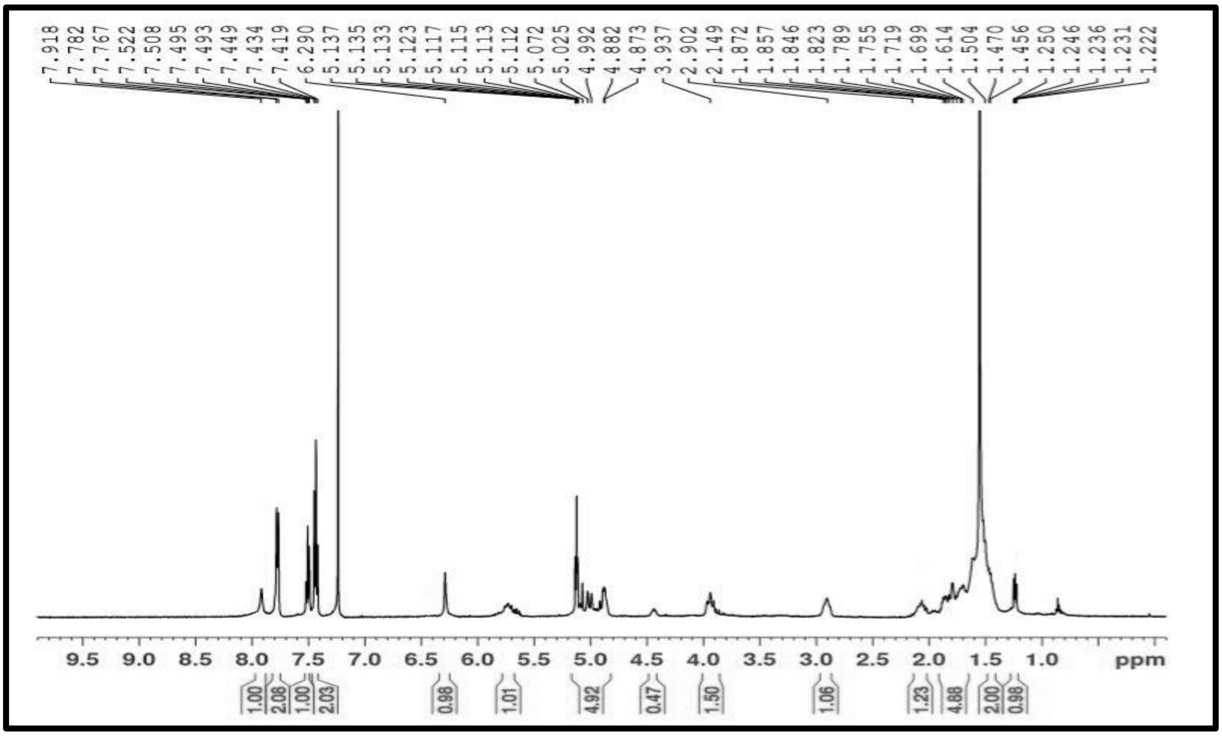

^{13}C-NMR

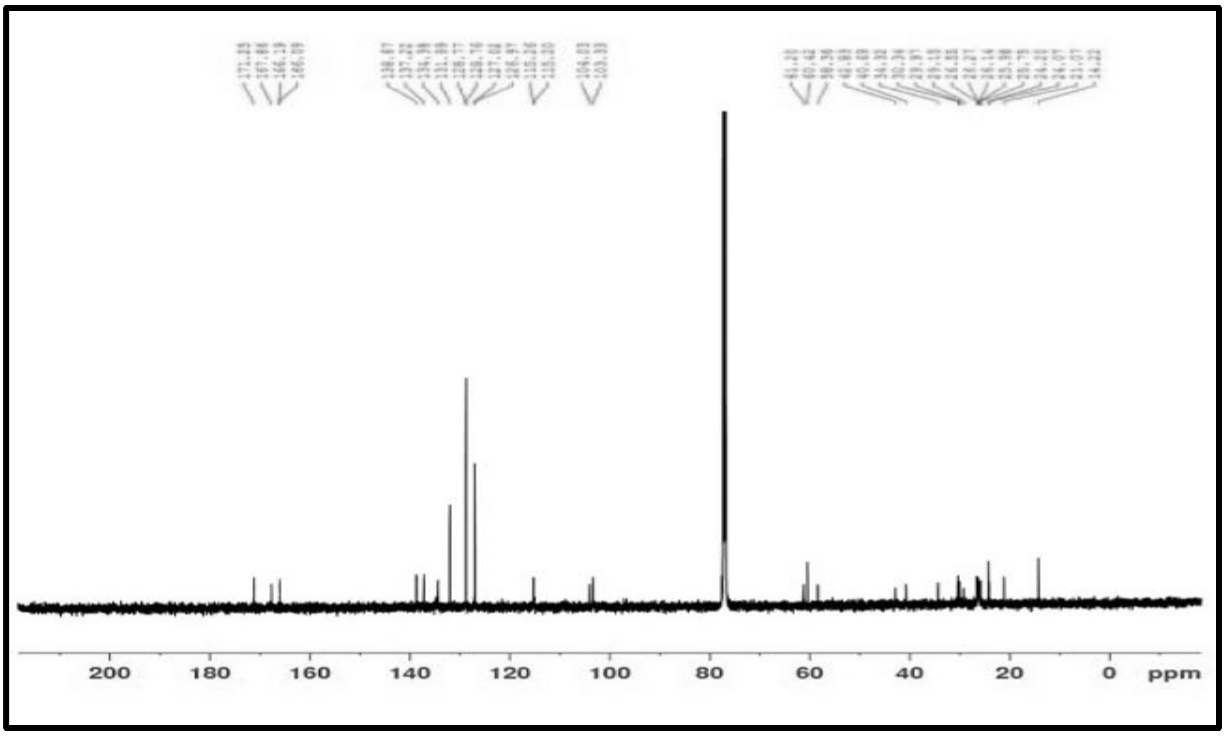

2-Phenyl-15-vinyl-3-oxa-1,7-diazaspiro[4.10]pentadec-1-en-6-one (**3i**)

^{1}H-NMR

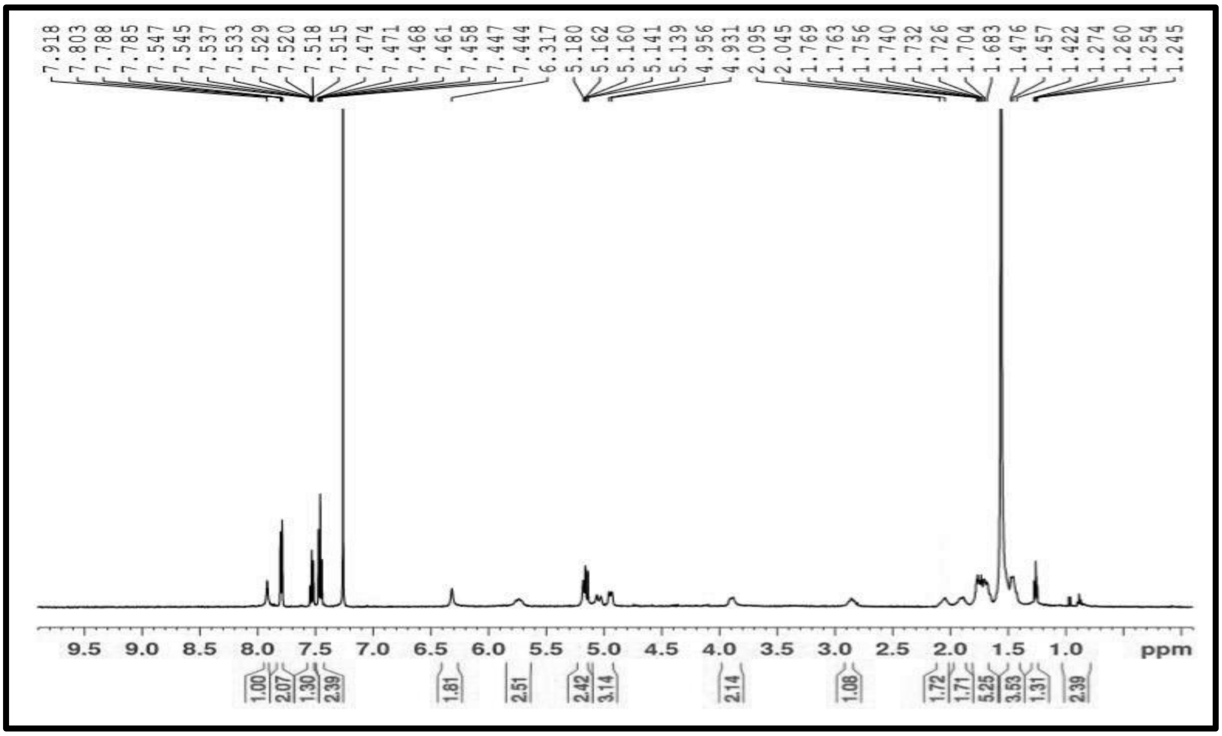

^{13}C-NMR

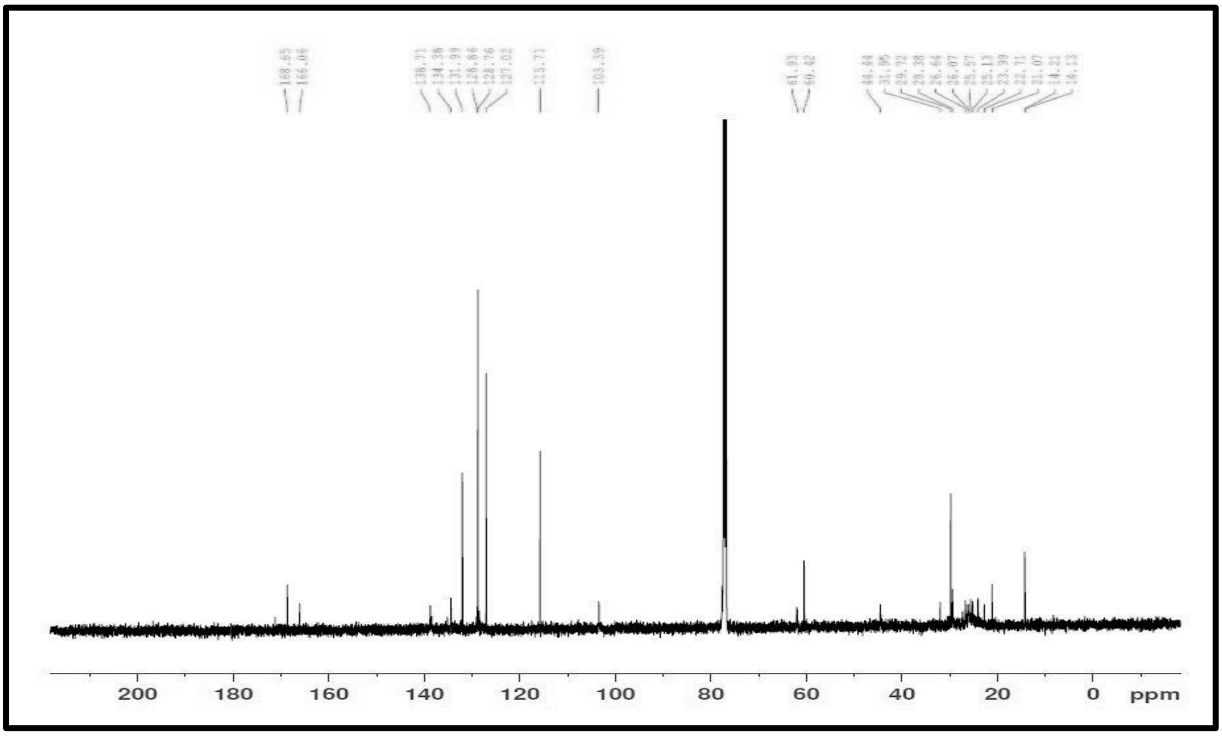

4-Allyl-*N*-benzyl-2-phenyl-4,5-dihydrooxazole-4-carboxamide (**3j**)

^{1}H-NMR

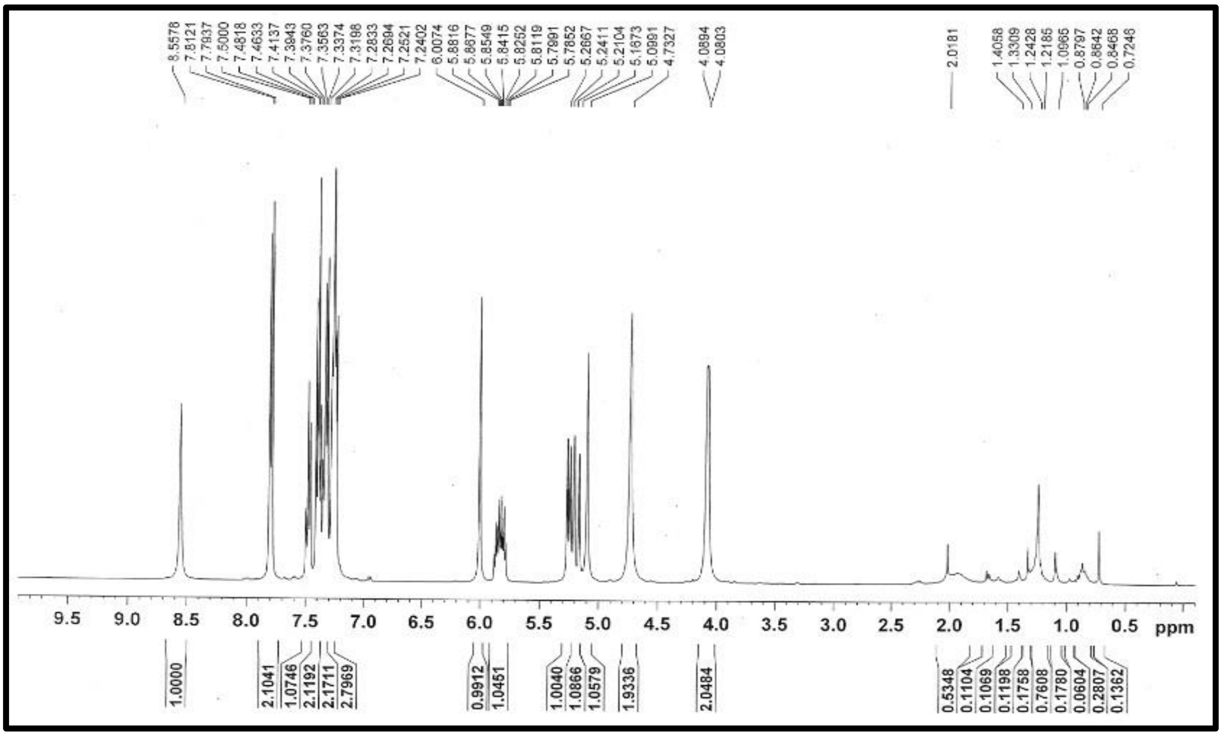

^{13}C-NMR

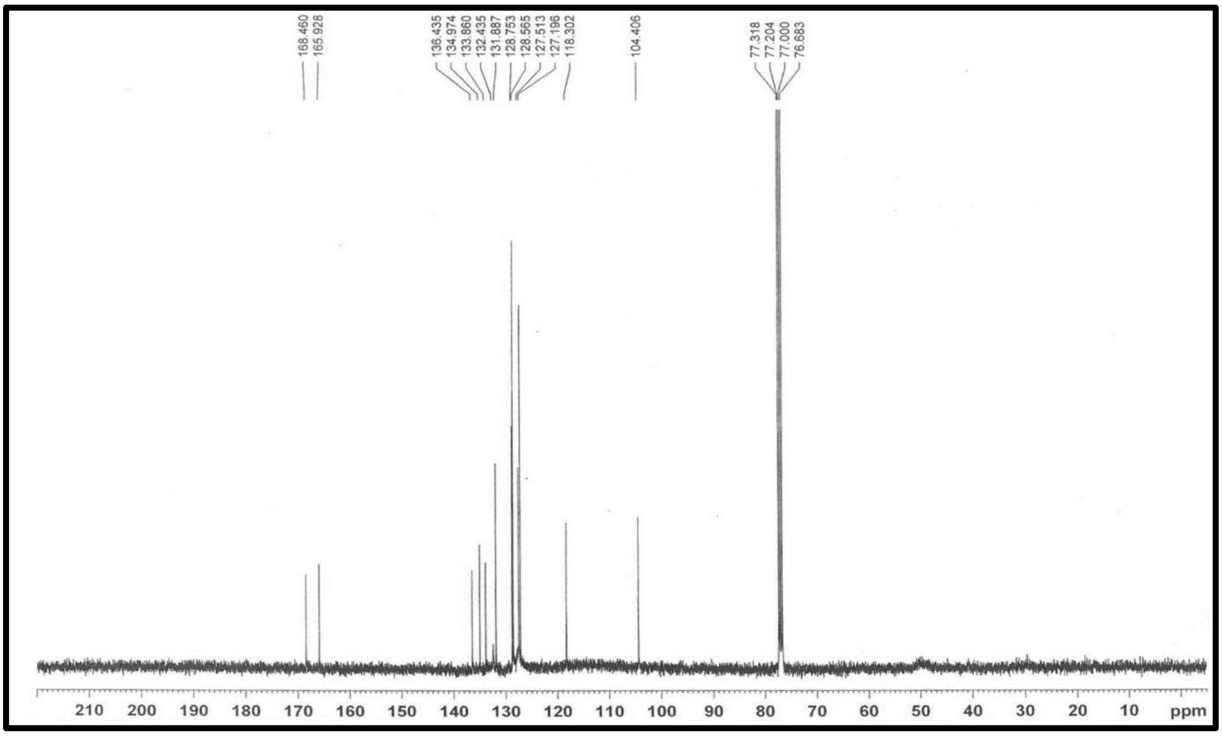

N-benzyl-4-cinnamyl-2-phenyl-4,5-dihydrooxazole-4-carboxamide (**3k**)

^{1}H-NMR

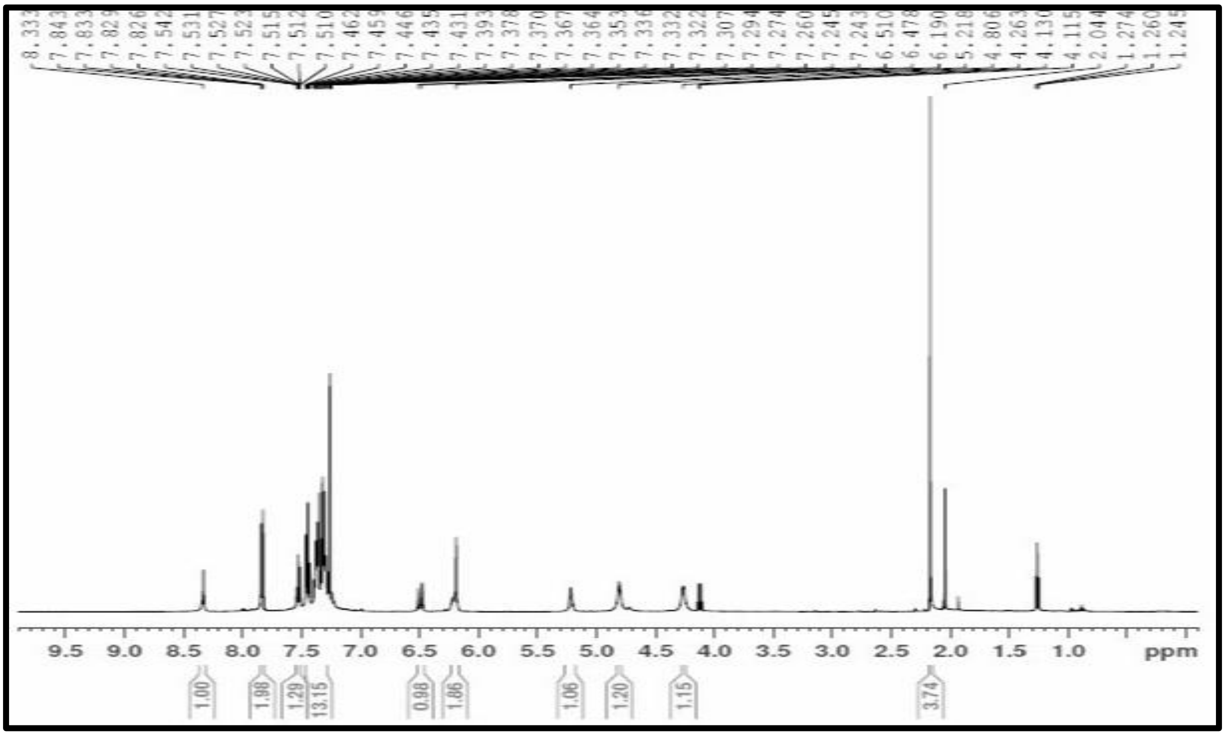

^{13}C-NMR

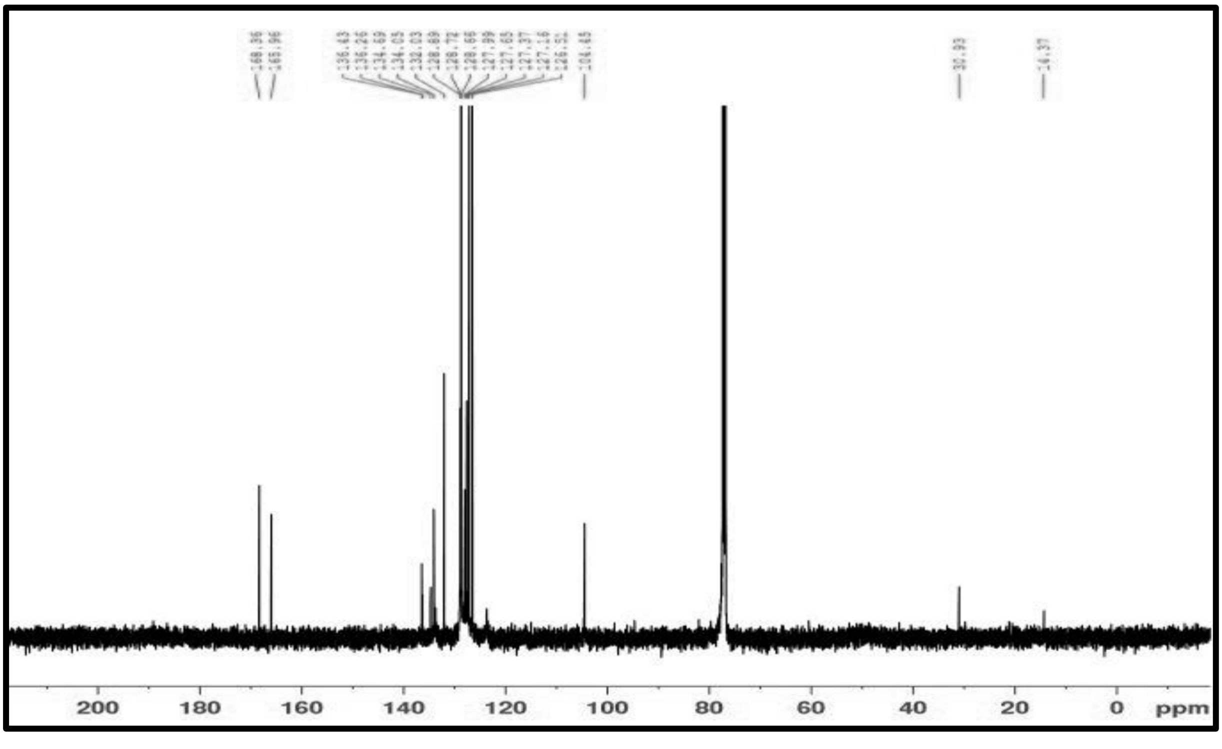

(*E*)-*N*-benzyl-4-(but-2-en-1-yl)-2-phenyl-4,5-dihydrooxazole-4-carboxamide (**3l**)

13H-NMR

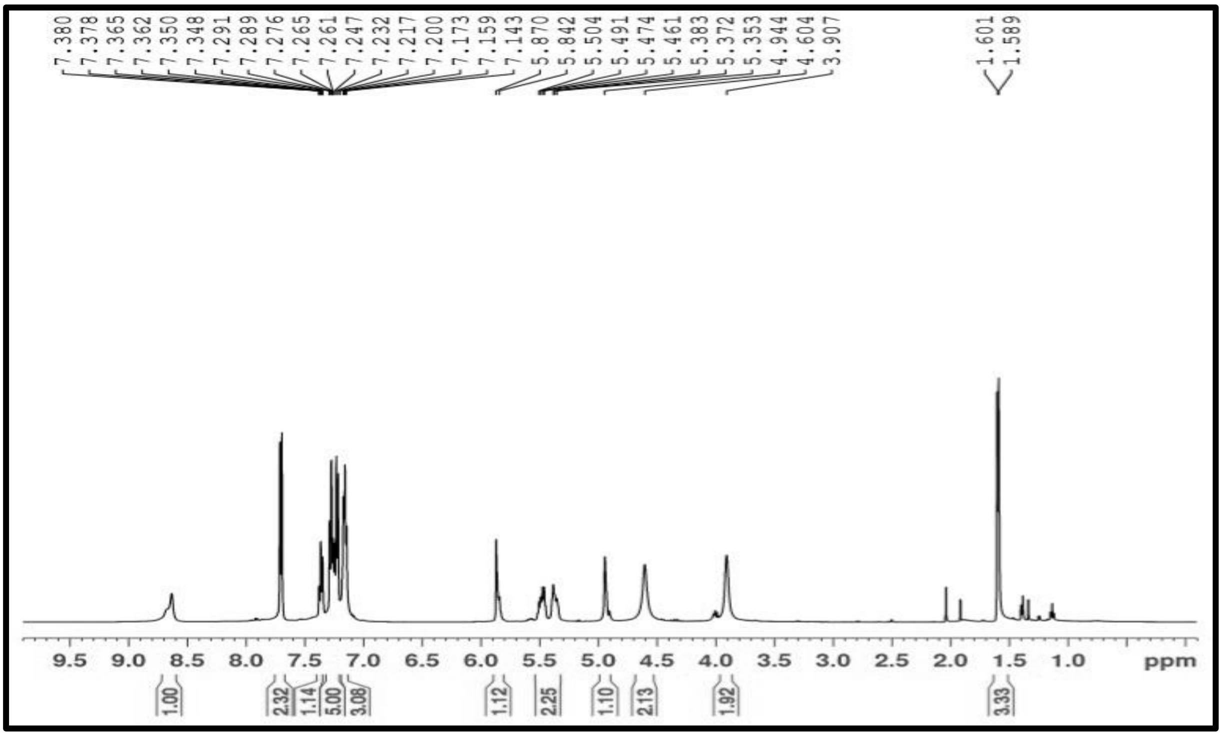

13C-NMR

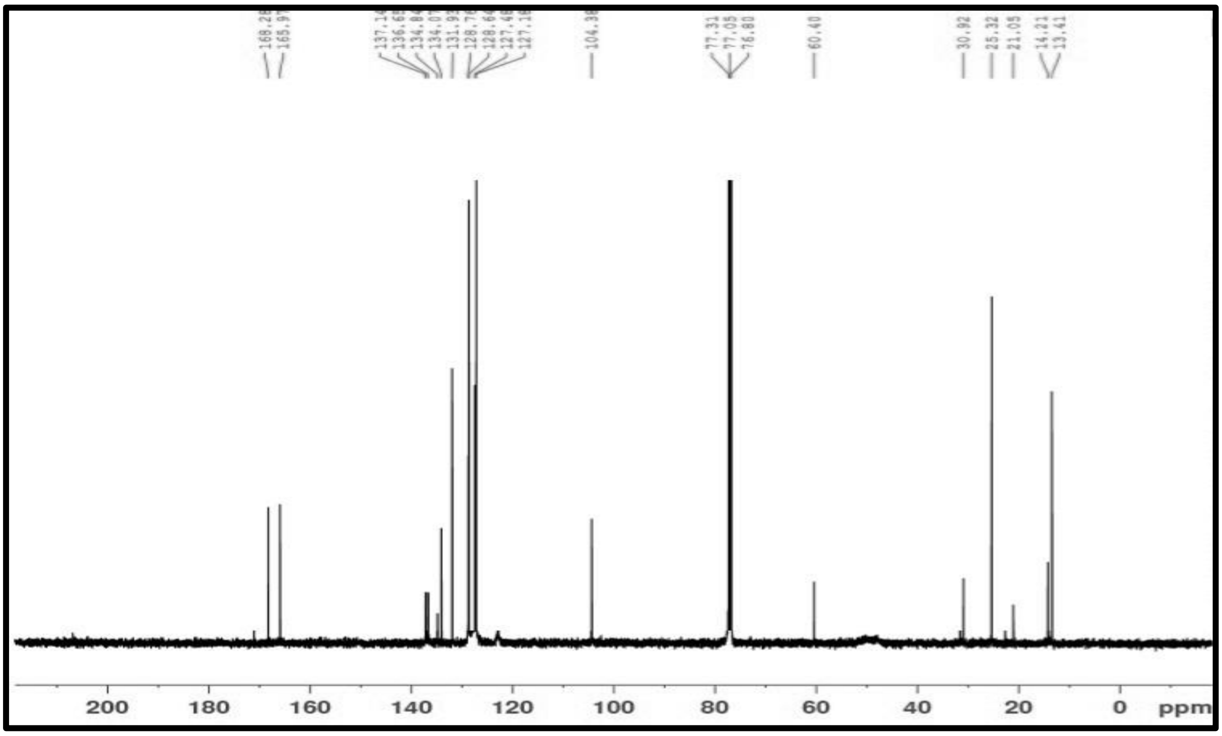

(*E*)-*N*-benzyl-4-(pent-2-en-1-yl)-2-phenyl-4,5-dihydrooxazole-4-carboxamide (**3m**)

^{1}H-NMR

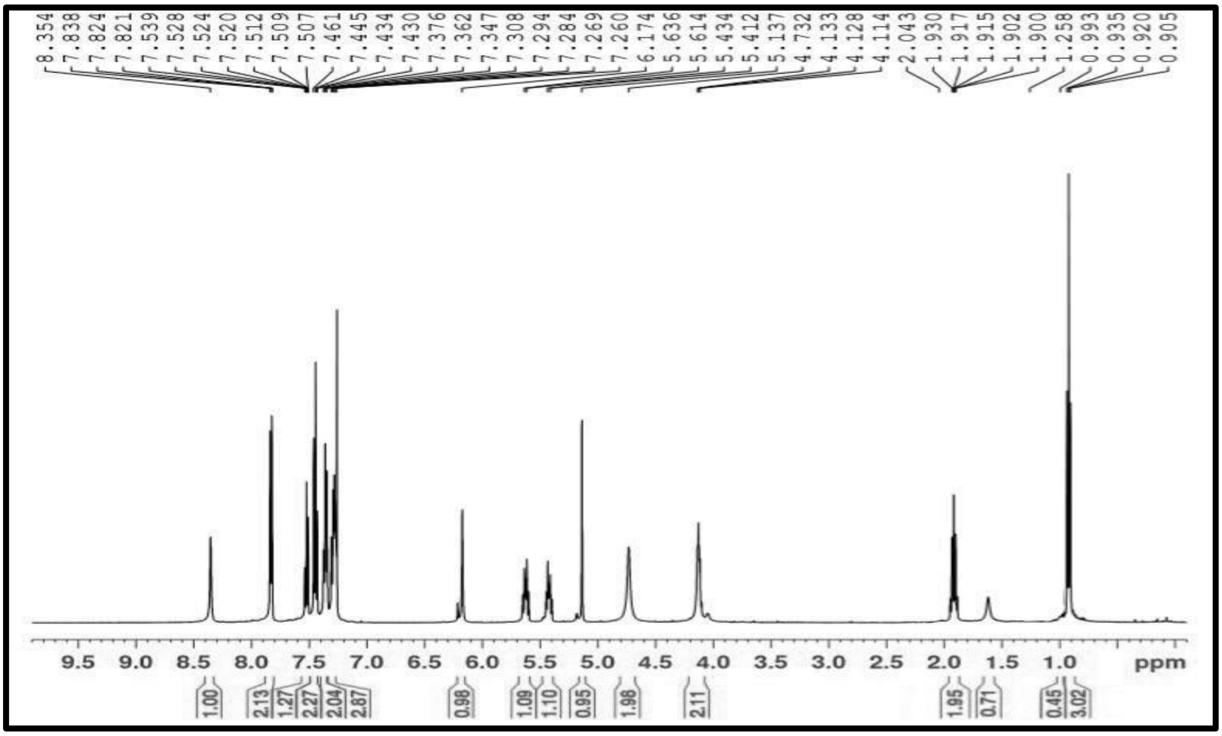

^{13}C-NMR

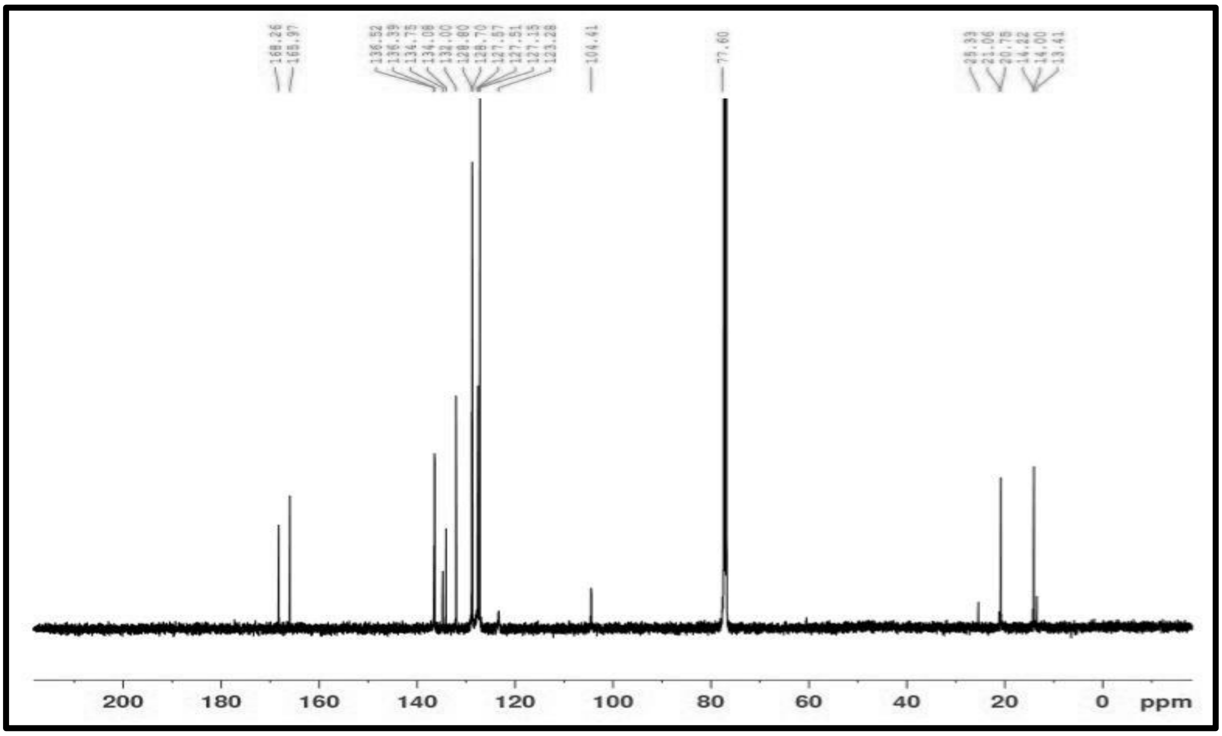

(*Z*)-*N*-benzyl-4-(pent-2-en-1-yl)-2-phenyl-4,5-dihydrooxazole-4-carboxamide (**3n**)

^{1}H-NMR

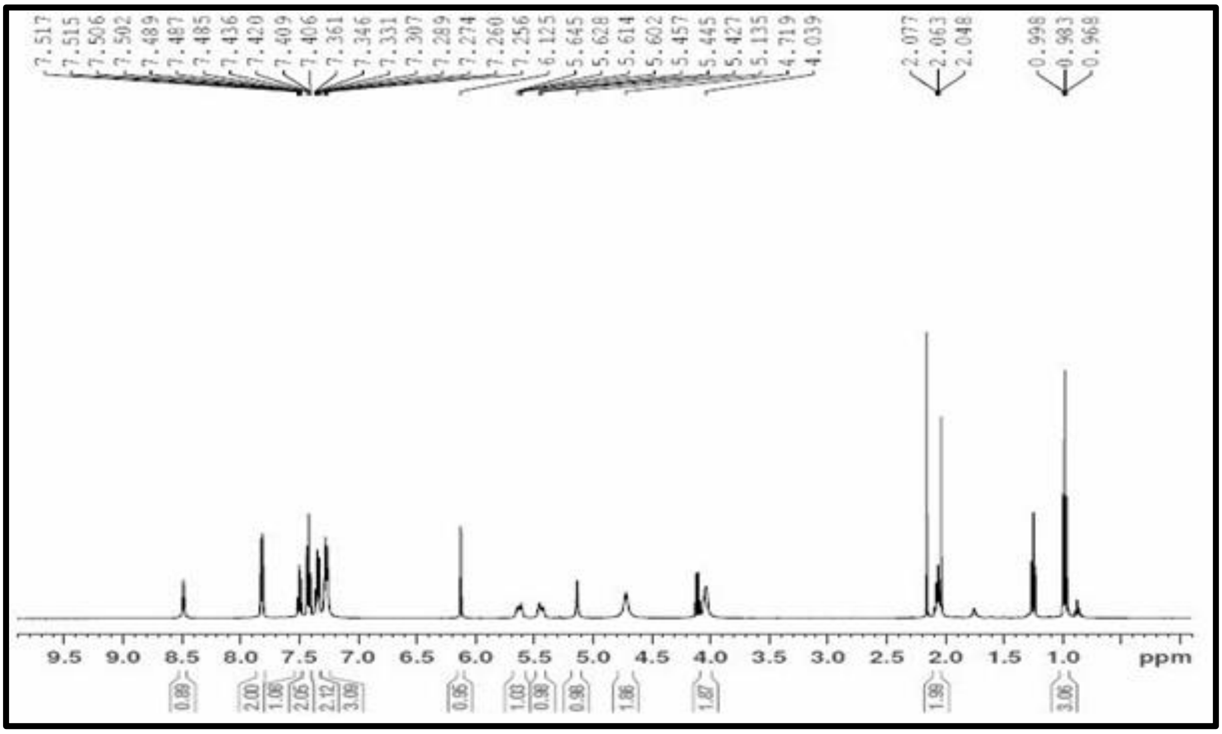

^{13}C-NMR

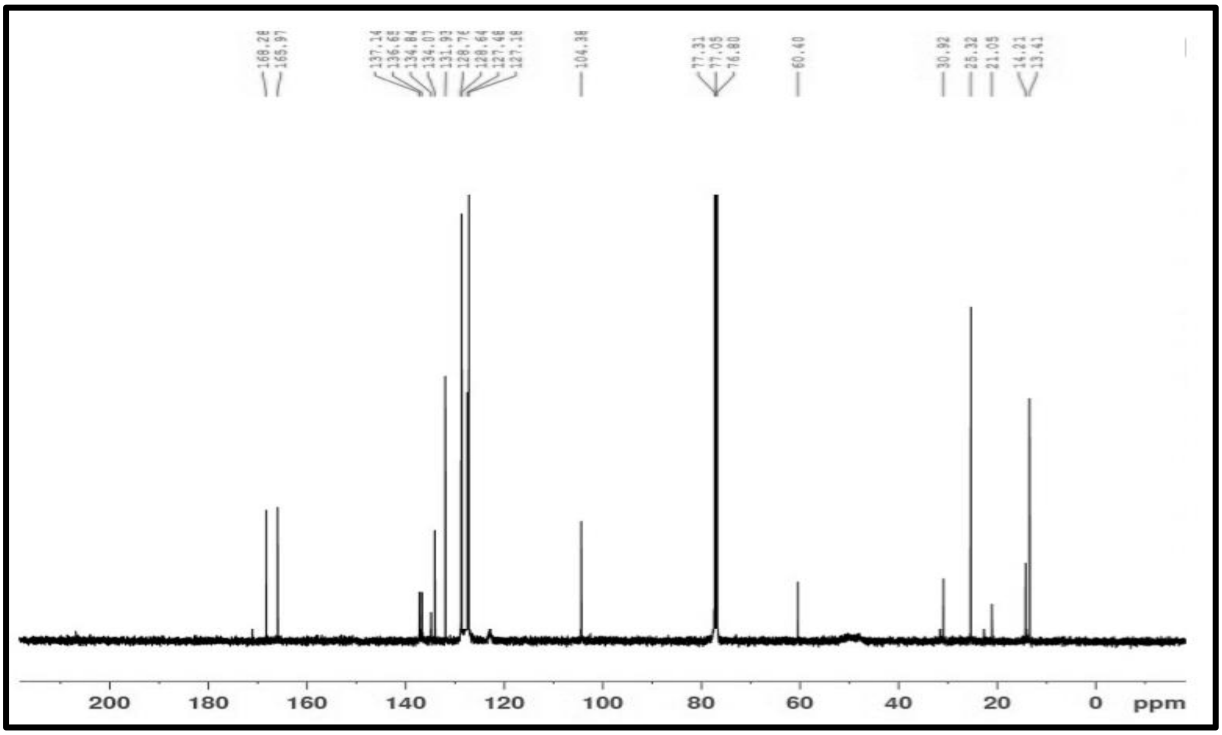

Supplement: Supplementary file 1 [file molecules-24-04495-s001.pdf]
